# Cellular senescence contributes to age‐dependent changes in circulating extracellular vesicle cargo and function

**DOI:** 10.1111/acel.13103

**Published:** 2020-01-21

**Authors:** Faisal J. Alibhai, Fievel Lim, Azadeh Yeganeh, Peter V. DiStefano, Tina Binesh‐Marvasti, Alyssa Belfiore, Lukasz Wlodarek, Dakota Gustafson, Sean Millar, Shu‐Hong Li, Richard D. Weisel, Jason E. Fish, Ren‐Ke Li

**Affiliations:** ^1^ Toronto General Hospital Research Institute Toronto General Hospital Toronto ON Canada; ^2^ Department of Laboratory Medicine and Pathobiology University of Toronto Toronto ON Canada; ^3^ Division of Cardiac Surgery Peter Munk Cardiac Centre Toronto General Hospital and University of Toronto Toronto ON Canada

**Keywords:** aging, extracellular vesicles, microRNA, plasma, senescence, senolytic

## Abstract

Extracellular vesicles (EVs) have emerged as important regulators of inter‐cellular and inter‐organ communication, in part via the transfer of their cargo to recipient cells. Although circulating EVs have been previously studied as biomarkers of aging, how circulating EVs change with age and the underlying mechanisms that contribute to these changes are poorly understood. Here, we demonstrate that aging has a profound effect on the circulating EV pool, as evidenced by changes in concentration, size, and cargo. Aging also alters particle function; treatment of cells with EV fractions isolated from old plasma reduces macrophage responses to lipopolysaccharide, increases phagocytosis, and reduces endothelial cell responses to vascular endothelial growth factor compared to cells treated with young EV fractions. Depletion studies indicate that CD63^+^ particles mediate these effects. Treatment of macrophages with EV‐like particles revealed that old particles increased the expression of EV miRNAs in recipient cells. Transfection of cells with microRNA mimics recapitulated some of the effects seen with old EV‐like particles. Investigation into the underlying mechanisms using bone marrow transplant studies revealed circulating cell age does not substantially affect the expression of aging‐associated circulating EV miRNAs in old mice. Instead, we show that cellular senescence contributes to changes in particle cargo and function. Notably, senolytic treatment of old mice shifted plasma particle cargo and function toward that of a younger phenotype. Collectively, these results demonstrate that senescent cells contribute to changes in plasma EVs with age and suggest a new mechanism by which senescent cells can affect cellular functions throughout the body.

## Graphical abstract

Senescent cells accumulate in the body with aging and alter circulating extracellular vesicle microRNA content. This can be replicated in young mice by inducing senescence using sub‐lethal irradiation. Conversely, removal of senescent cells in aged mice by senolytic treatment restores circulating extracellular vesicle microRNA expression to that of a younger phenotype.

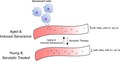

## 1 INTRODUCTION

Aging is associated with a decline in the function of a number of organ systems which ultimately contributes to increased risk of developing age‐related diseases (Lopez‐Otin, Blasco, Partridge, Serrano, & Kroemer, 2013). Although much attention has been given to how aging affects tissue structure and function, less is known about how aging affects circulating factors. Investigation of circulating factors has been primarily focused on biomarker discovery for disease diagnosis and risk stratification. However, more recent studies have demonstrated that circulating factors that change with age can affect tissue homeostasis. In a landmark study, Conboy and colleagues demonstrated that exposure of aged mice to young circulating factors improved skeletal muscle progenitor cell function (Conboy et al., 2005). Since this initial discovery, there have been a number of studies demonstrating the ability of young circulating factors to improve the function of aged tissues (Conboy & Rando, 2012). However, there are also changes in the aged host's circulation which adversely affect tissue function/repair. For example, Rebo and colleagues demonstrated that a single blood exchange between young and old mice led to rapid inhibition of skeletal muscle regeneration in young mice (Rebo et al., 2016). Therefore, there is a need to better define the factors that change in the aged circulation to understand the pathophysiology of aging.

Extracellular vesicle (EV) is a broad term used to describe membrane encapsulated vesicles that range in size from 30 to 1,000 nm and arise from different modes of secretion. Extracellular vesicles are secreted by cell types throughout the body into the extracellular environment or bodily fluids such as the blood and urine where they carry a number of factors including microRNAs (miRNAs) and proteins (Alibhai, Tobin, Yeganeh, Weisel, & Li, 2018; Yanez‐Mo et al., 2015). Changes in plasma EV content reflect changes in cellular function in a number of disease states. For example, changes in circulating EV cargo have been observed in diabetes, postmyocardial infarction, and in cancer (Jansen, Nickenig, & Werner, 2017; Schwarzenbach, 2015). Extracellular vesicles also play a role in inter‐cellular communication as they are capable of transferring their cargo to influence recipient cell function. Given their potential for regulating cellular function, EVs have been suggested to be key mediators of aging (Robbins, 2017; Takasugi, 2018). Despite this interest, how plasma EVs change with age and the underlying mechanisms that contribute to these changes are poorly understood.

Here, we investigate the changes that occur in plasma EVs during aging. We examine how aging affects plasma EV concentration, size, cargo, and function. Using bone marrow transplant experiments, we investigate the role of aging circulating cells in regulating plasma EV miRNA expression. Furthermore, using in vitro and in vivo models of senescence as well as senolytic treatment of aged mice we examine the contribution of senescent cells to plasma EVs. Our study suggests a key role of cellular senescence in regulating circulating EV miRNA cargo and function in aged mice.

## 2 RESULTS

### 2.1 Aging affects plasma EV concentration and size

**Figure 1:**
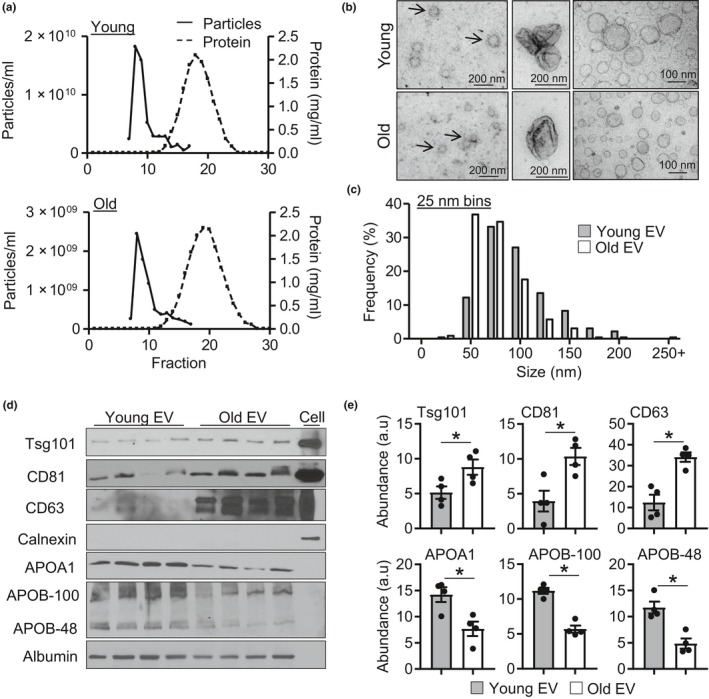
Aging alters plasma EV‐like particle concentration and size. (a) Size exclusion chromatography isolation of plasma EV enriched fractions from young (top) and old (bottom) plasma. (b) Representative transmission electron microscopy (TEM) images of particles isolated from young and old plasma. (c) Quantification of particle size distribution in 25 nm bins from TEM images, *n* = 3/group. (d) Representative Western blots images and (e) quantification, *n* = 4/group, *p < .05. All values are mean ± *SEM*

Plasma EVs were isolated from young (3 month) and old (18‐21 month) mice using size exclusion chromatography (SEC) which efficiently separates plasma particles from plasma proteins (Figure 1a). Based on the peak particle count and separation from plasma protein, fractions 7–10 were collected for experiments. Examination of isolated particles by transmission electron microscopy (TEM) revealed particles with diverse sizes (<300 nm in diameter) and morphologies, including the expected “cup‐shaped” morphology in both preparations (Figure 1b). Quantification of particle size from TEM images revealed a greater number of smaller particles in old versus young EV fractions (Figure 1c). To further quantify changes in particle size and concentration, we used nanoparticle tracking analysis (NTA) which measures the rate of particle Brownian motion in solution to determine size and uses the number of particles tracked to determine concentration. Nanoparticle tracking analysis revealed a significant reduction in plasma particle concentration and smaller mean particle size in old versus young EV fractions (Figure S1a–c). A decline in particle concentration with aging has been previously shown with human plasma using a precipitation‐based method (Eitan et al., 2017); thus, we next assessed whether old murine plasma also shows reduced particle count using this approach. Lower particle count in old versus young plasma was also observed using the precipitation method; however, the amount of co‐isolated protein was substantially higher compared to fractions collected using SEC, suggesting reduced purity (Figure S1e–i). Thus, we used SEC‐purified vesicles for this study.

As NTA cannot distinguish EVs from other particles such as lipoproteins, we characterized isolated plasma particles by flow cytometry and Western blotting. Surprisingly, examination of CD63, an EV marker, using flow cytometry revealed significantly increased fluorescence in old versus young EV fractions (Figure S1d). Western blotting similarly demonstrated significantly greater levels of TSG101, CD81, and CD63 in old versus young EV fractions, suggesting increased EV levels in old plasma (Figure 1d,e). Assessment of plasma contaminants revealed that old EV fractions had significantly less APOA1, APOB‐100, and APOB‐48 compared with young fractions (Figure 1d,e). Reduced lipoprotein content may be responsible for the lower particle counts by NTA as chylomicrons which carry APOB represent a substantial number of plasma particles (Sodar et al., 2016). Albumin levels were similar between young and old EV fractions (Figure S1i). Importantly, neither EV fractions contained organelle contaminants indicated by a lack of calnexin signal. Collectively, these data demonstrate that although circulating particle count is lower; EV levels are significantly elevated in old plasma. As characterization of our EV fractions indicate that SEC leads to co‐isolation of plasma factors, the collected fractions used are referred to as EV enriched fractions and particles as EV‐like particles throughout this study.

### 2.2 Old plasma EVs alter cellular responses to stimuli

**Figure 2:**
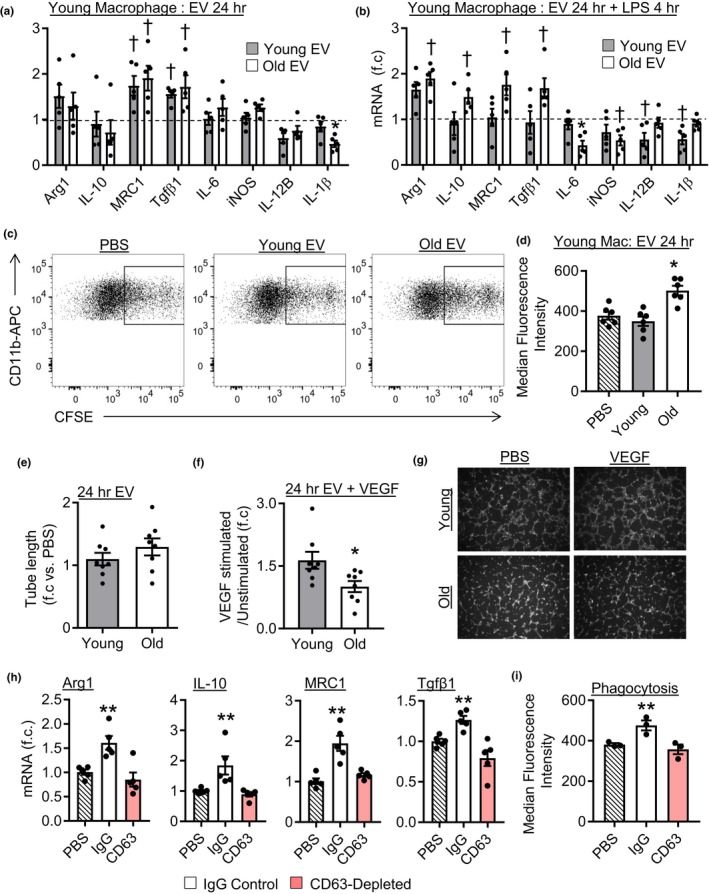
Aging alters plasma EV‐like particle function. (a) Gene expression in young macrophages treated with plasma EVs for 24 hr; data are relative to PBS‐treated cells (dashed line), *n* = 4–5/group. (b) Gene expression in young macrophages treated with EVs for 24 hr then LPS for 4 hr. Expression is relative to cells treated with PBS for 24 hr then LPS for 4 hr (dashed line), *n* = 5/group, ^†^ *p* < .05 versus PBS‐treated cells, *p < .05 versus all other groups. (c) Representative flow cytometry images of macrophage phagocytosis and (d) quantification, *n* = 6/group, *p < .05 versus all groups. (e) Quantification of tube formation in HUVECs treated with plasma EVs. (f) Quantification of tube formation of cells treated with plasma EVs and stimulated with VEGF. (g) Representative tube formation images. *n* = 8/group, *p < .05. (h) Young macrophages treated with old EVs (IgG or CD63 depleted) for 24 hr then LPS for 4 hr, *n* = 5/group, **p < .05 versus all groups. (i) Phagocytic activity of young macrophages treated with old EVs (IgG or CD63 depleted), *n* = 3/group, **p < .05 versus all groups. All values are mean ± *SEM*

Next, we investigated whether aging affects the function of isolated plasma EV enriched fractions. The functional effects of young and old EV fractions were tested using two cell types that interact with circulating EVs, macrophages and endothelial cells. First, we examined how EV fractions affected the expression of activation markers in unstimulated macrophages including polarization markers arginase 1 (Arg1), mannose Receptor C‐Type 1 (MRC1), transforming growth factor β‐1 (Tgfβ1), and inducible nitric oxide synthase (iNOS) as well as cytokine expression. Treatment of young unstimulated macrophages with young or old EV fractions increased the expression of MRC1 and Tgfβ1 compared to PBS‐treated cells (Figure 2a and Figure S2). Although old EV fractions reduced IL1β expression compared to young EVs, the overall trend of basal gene expression was similar between young and old EV‐treated cells and many of these cytokines had low expression in unstimulated cells. Thus, we next investigated whether young or old EV enriched fractions can affect cellular responses to activation. Following stimulation of young macrophages with LPS, old EV‐treated cells had increased Arg1, IL10, MRC1, and Tgfβ1 as well as reduced IL‐6 and iNOS expression compared to PBS‐treated cells (Figure 2b). In contrast, young macrophages treated with young EVs had significantly reduced expression of IL1β and IL12B compared to PBS‐treated cells (Figure 2b and Figure S2). Next, we examined whether EV fractions can also affect old macrophage responses to LPS. Interestingly, the effects on old cells were different than those observed in young cells. In unstimulated cells, old EV fractions reduced the expression of IL‐1β, IL‐12B, and IL‐10 compared to PBS‐treated cells (Figure S3). Young EV fractions also reduced expression of IL‐1β, but did not affect IL‐12B, IL‐10, MRC1 or Tgfβ1 expression. In LPS‐stimulated cells old EVs reduced expression of IL‐1β, IL‐12, IL‐6, and IL‐10 as well as increased Arg1 expression compared to PBS‐treated cells (Figure S3). Young EVs did not affect gene expression in old LPS‐stimulated cells (Figure S3).

To further understand how EV enriched fractions affect macrophage function, we examined the ability of macrophages to perform cell phagocytosis following EV treatment. CFSE‐stained apoptotic cells were added to cultured macrophages previously treated with PBS, young EVs, or old EV fractions. Examination of CFSE^+^ macrophages by flow cytometry demonstrated similar levels of phagocytosis in PBS and young EV‐treated cells. However, old EV fractions significantly increased the number of CFSE^+^ macrophages compared to PBS and young EV fractions treated cells, indicating increased phagocytic activity (Figure 2c,d). Enhanced phagocytosis ability is consistent with the promotion of an immunomodulatory phenotype by old EVs. Examination of plasma EV‐like particle induced phagocytosis in old cells revealed that young and old EVs had no effect (Figure S4b).

Next, we examined the effect of young and old EV fractions on endothelial function using tube formation as an index of angiogenic function. No difference in tube formation was observed between cells treated with young or old EV fractions for 24 hr in the absence of VEGF (unstimulated) (Figure 2e). However, we found that old EV fractions blocked endothelial responses to vascular endothelial growth factor (VEGF). In PBS or young EV‐treated cells, VEGF stimulation led to a ~1.5‐ to 1.6‐fold increase in tube formation compared to unstimulated cells (Figure 2f,g and Figure S4c). In contrast, cells treated with old EV fractions did not exhibit an increase in tube formation following VEGF stimulation (Figure 2f,g). Collectively, these results demonstrate that old EV fractions alter macrophage responses to LPS in young and old cells, increase macrophage phagocytosis in young cells, and reduce endothelial responses to VEGF. In contrast, young EV fractions reduce pro‐inflammatory cytokine expression following LPS stimulation only in young cells.

While our plasma EV preparations were relatively free of protein contamination, we sought to demonstrate that the observed functional effects were due to EVs, rather than contaminants. We therefore depleted CD63^+^ particles from isolated fractions using anti‐CD63‐coated beads and compared the functional effects to IgG bead (control) treated fractions. First, we confirmed the capture of CD63^+^ particles using anti‐CD63‐coated beads and that IgG beads did not capture CD63^+^ particles (Figure S1j). Young macrophages were treated with IgG or CD63‐depleted EV fractions after which the response to LPS was assessed. Cells treated with EV fractions incubated with IgG beads exhibited similar profiles as described above; old EVs increased Arg1, IL‐10, MRC1, and Tgfβ1 expression and reduced IL‐6 and iNOS expression compared to PBS‐treated cells. However, in CD63‐depleted fractions old EV fractions no longer stimulated expression of Arg1, IL‐10, MRC1, and Tgfβ1 or reduced IL‐6 and iNOS expression, as expression was similar to PBS‐treated cells (Figure 2h and Figure S5a). In contrast, the effects observed with young EV fractions were similar between IgG and CD63 treated fractions as both reduced the expression of pro‐inflammatory cytokines compared to PBS‐treated cells (Figure S5b). This indicates that the effects observed with young EV preparations may be due to other non‐EV co‐isolated factors. Lastly, we examined whether depletion of CD63 altered the ability of old EV fractions to stimulate macrophage phagocytosis. Cells treated with old EV fractions incubated with control beads exhibited an increase in phagocytic activity, consistent with our previous results. Following CD63 depletion old EV fractions no longer simulated phagocytosis, indicating that CD63^+^ particles act to increase macrophage phagocytosis (Figure 2i). CD63 depletion did not affect young EVs as both IgG and CD63 treated fractions did not affect phagocytic function (Figure S5c,d). Together these data using depleted fractions indicate that CD63^+^ particles are key effectors in old EV fractions.

### 2.3 Aging alters plasma EV miRNA expression

**Figure 3:**
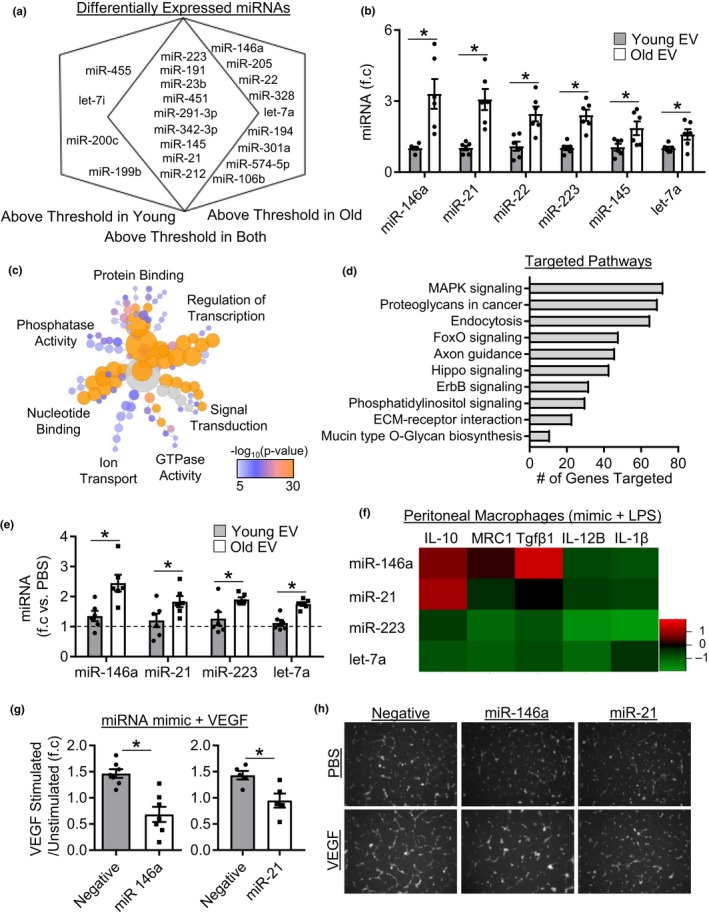
Aging alters plasma EV‐like particle Cargo. (a) Venn diagram showing differentially expressed miRNAs between young and old EVs, *n* = 4 samples per group. (b) Real‐Time PCR validation of differentially expressed miRNAs in an independent sample set, *n* = 6/group, *p < .05. (c) Bioinformatics analysis of the molecular function of miRNAs upregulated in old EVs and (d) predicted KEGG pathways targeted. (e) MiRNA expression in macrophage treated with either young or old plasma EVs. Expression is relative to PBS‐treated cells (dashed line). *n* = 6/group, *p < .05 old EV versus young EV. (f) Log_2_ gene expression in macrophages transfected with miRNA mimics then stimulated with LPS for 4 hr, *n* = 5/group. (g) Quantification of tube formation in stimulated/unstimulated cells in control, miR‐146a, and miR‐21 transfected cells, *n* = 5‐7/group. (h) Representative tube formation images. All values are mean ± *SEM*

To investigate whether aging impacts plasma EV‐like particle cargo and whether this cargo may contribute to changes in function, we screened miRNA expression in young and old EV fractions. Overall, 65 miRNAs were detectable above threshold in young and old EV fractions, with 52 present in both, nine only in old EVs, and four only in young EVs (Table S2). Moreover, of the 52 miRNAs that were expressed in both young and old EVs we identified nine miRNAs with significantly different expression between young and old EV fractions (Figure 3a and Figure S6a). We were interested in pursuing miRNAs that were elevated in old EVs, as this might help explain why old EVs acquire an inhibitory phenotype. Thus, we validated differentially expressed miRNAs in a second independent sample set by qPCR and confirmed significantly increased expression of miR‐146a, miR‐21, miR‐22, miR‐223, miR‐145, and let‐7a in old EVs compared to young EVs (Figure 3b). We also validated miRNAs with greater expression in young EV fractions, as shown by significantly greater miR‐212 and miR‐455 expression in young EVs (Figure S6b). To understand the functional impact changes in miRNA expression may have, we assessed the predicted molecular function and pathways targeted by differentially expressed miRNAs. Gene ontology of predicted mRNA targets was clustered to reveal the enriched molecular functions of target genes. MicroRNAs upregulated in old mice primarily targeted genes involved in the regulation of transcription, nucleotide binding, and signal transduction (Figure 3c). Further analysis into the pathways targeted revealed that target genes are involved in a number of signaling pathways, with the most number of gene targets associated with MAPK signaling (Figure 3d). We next investigated whether changes in EV miRNA cargo contribute to functional changes. First, we determined whether plasma EV‐like particles were capable of transferring miRNAs. Young and old particles were transfected with cel‐miR‐39, a miRNA not expressed in mice, macrophages treated, and expression examined (Figure S5e). Both young and old EV‐like particles were capable of transferring cargo, and expression levels were similar between 6 and 24 hr indicating rapid uptake of vesicles. Interestingly, cel‐miR‐39 expression was higher in cells treated with transfected old EV‐like particles. However, this may be related to the increased number of EVs in old fractions. Further examination of miRNA transfer revealed that macrophages treated with old EV fractions had significantly greater levels of miR‐146a, miR‐21, miR‐223, and let‐7a compared to cells treated with young EV fractions (Figure 3e). However, young EV‐like particles are also capable of cargo of transfer as macrophages treated with young EV‐like particles exhibited greater expression of miR‐212 and miR‐455 compared to old EV‐treated cells (Figure S6c). Next, we examined the functional effects of these miRNAs. Macrophages were transfected with miRNA mimics after which the cells were stimulated with LPS and gene expression examined. Average log2 mRNA expression in mimic versus negative control transfected cells is shown in Figure 3f, and fold change values with individual data points are shown in Figure S6d–g. No single miRNA recapitulated the effect observed in cells pretreated with old EV fractions. However, similar to old EVs miR‐146a and miR‐21 stimulated the expression of immunomodulatory genes Tgfβ1 and IL10, respectively. In contrast, miR‐223 and let‐7a reduced the induction of both pro‐inflammatory and immunomodulatory genes in LPS‐stimulated macrophages (Figure 3f). We also tested whether miR‐146a and miR‐21 could block endothelial cell responses to VEGF. Cells transfected with negative control showed a ~ 1.5‐fold increase in tube formation in response to VEGF treatment (Figure 3g,h). However, cells transfected with miR‐146a or miR‐21 mimics did not increase tube formation in response to VEGF treatment (Figure 3g,h). These data demonstrate that miR‐146a and miR‐21 in part recapitulate the functional effects of old EV‐like particles.

### 2.4 Effect of bone marrow (BM) and circulating cell age on plasma EV miRNA expression

**Figure 4:**
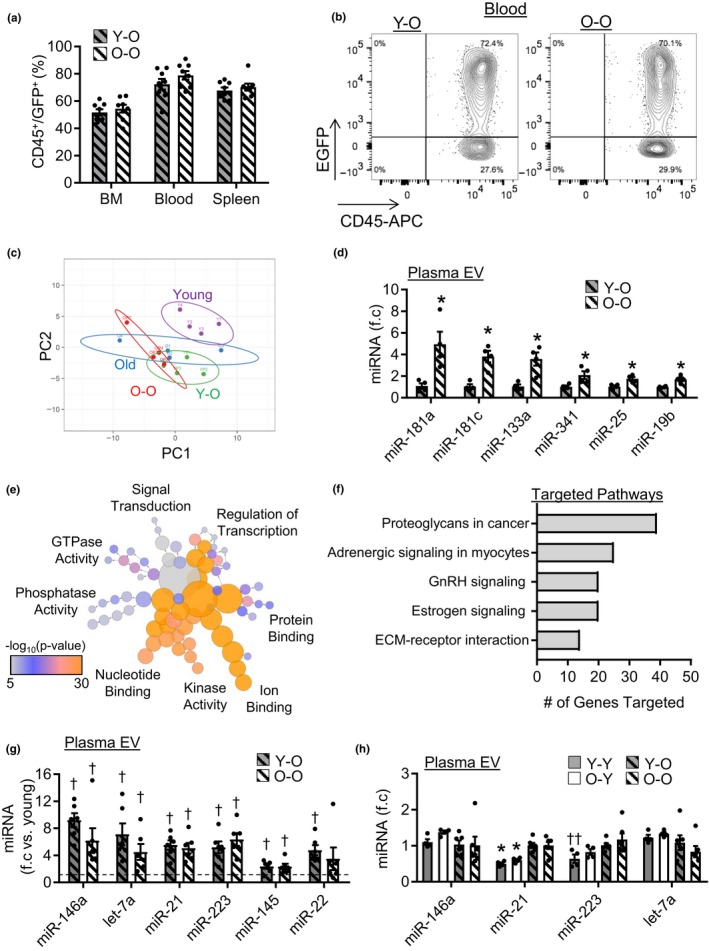
Effect of bone marrow (BM) and circulating cell age on EV miRNA expression. (a) Quantification young and old reconstitution in aged mice, *n* = 8/group. (b) Representative flow cytometry images of donor CD45/GFP^+^ cells in the blood. (c) Principle component analysis of miRNA expression in plasma EVs from young, old, YO and OO mice. (d) Differentially expressed miRNAs in plasma EVs from YO and OO mice identified by miRNA qPCR array, *n* = 4/group, *p < .05. (e) Bioinformatics analysis of the molecular function of miRNAs differentially expressed between YO and OO mice and (f) predicted KEGG pathways targeted. (g) Expression of age‐associated miRNAs in YO and OO plasma EVs. Groups are relative to young plasma EV miRNA expression (dashed line), *n* = 6/group, ^†^ *p* < .05 versus young plasma EVs. (h) Plasma EV miRNA expression in YY, OY, YO, and OO mice, *n* = 4–6/group, *p < .05 versus YO and OO, and ^††^ *p* < .05 versus OO. All values are mean ± *SEM*

Plasma EVs have been suggested to arise, in part, from circulating cells (Wen et al., 2017). Moreover, miR‐21 and miR‐146a are classically associated with inflammation (Mann et al., 2017; Sheedy, 2015); thus, changes in miRNA expression may reflect increased activation of circulating cells in old mice. We therefore examined whether the age of bone marrow (BM) cells and their circulating progeny contribute to changes in plasma EVs. Old mice were lethally irradiated and reconstituted with either young (YO; old mice given young BM) or old (OO; old mice given old BM) GFP^+^ BM cells, after which circulating EVs were examined 3 months later. Both young and old BM transplants successfully reconstituted recipient animals, and these animals exhibited similar levels of donor cell chimaerism in the BM, blood, and spleen as determined by the percentage of GFP^+^/CD45^+^ cells (Figure 4a,b and Figure S7). We found that reconstitution of aged mice with young BM did not shift plasma EV‐like particle miRNA profiles toward a young phenotype as miRNA profiles clustered closer to old particles rather than with young (Figure 4c). Further examination of EV miRNAs in YO and OO mice revealed 70 miRNAs that were detectable in both samples. Analysis of differentially expressed miRNAs revealed significantly increased expression of miR‐181a, miR‐181c, miR‐133a, miR‐341, miR‐25, and miR‐19b in OO versus YO mice (Figure 4d). Examination of the predicted molecular functions and pathways targeted by differentially expressed miRNAs revealed that miRNAs with increased expression in OO mice targeted mRNAs primarily involved in ion binding, kinase activity, nucleotide binding (Figure 4e). Moreover, the predicted KEGG pathways were distinct from those predicted to be targeted by miRNAs which changed with aging (Figure 4f). Notably, miRNAs which differed between YO and OO mice appear to be restricted to animals undergoing reconstitution, as old mice did not exhibit changes in the expression of these miRNAs compared to young mice (Figure S8a). Interestingly, EV miRNA markers that were elevated with aging remained elevated in old reconstituted mice compared to young mice, regardless of BM donor age (Figure 4g). It is possible that old mice have surpassed some threshold and that reconstitution with young BM does not affect EV miRNA expression. Therefore, we reconstituted young mice with either young (YY) or old (OY) BM and assessed plasma EV fractions 3 months later (Figure 4h). We found a significant effect of recipient mouse age, as expression of miR‐21 was significantly lower in YY and OY mice compared with YO and OO (Figure 4h). Moreover, miR‐223 expression was partially affected by donor age as levels in YY were significantly lower compared to OO mice, whereas OY levels were not. In contrast, miR‐146a and let‐7a expression was comparable among all four groups. This suggests that the transplant procedure may also impact the expression of these miRNAs, as expression in young mice was similar to old mice following reconstitution. To further determine whether circulating cells secrete EVs with different miRNA expression related to aging, we assessed young and old peripheral blood mononuclear cells in vitro. Consistent with our findings, expression was similar in EVs isolated from young and old PBMCs (Figure S8b). Collectively, these data demonstrate that although changes in miRNA expression were observed between YO and OO mice, these changes appear to be restricted to mice undergoing BMT. Moreover, while these data support that the recipient and donor age can influence EV miRNA expression these differences were relatively small, and donor age effect was limited to only miR‐223. Therefore, donor age and by extension circulating cell age appear to have a minor effect on the miRNAs assessed in this study.

### 2.5 Induction of senescence leads to changes in circulating EV miRNA expression

**Figure 5:**
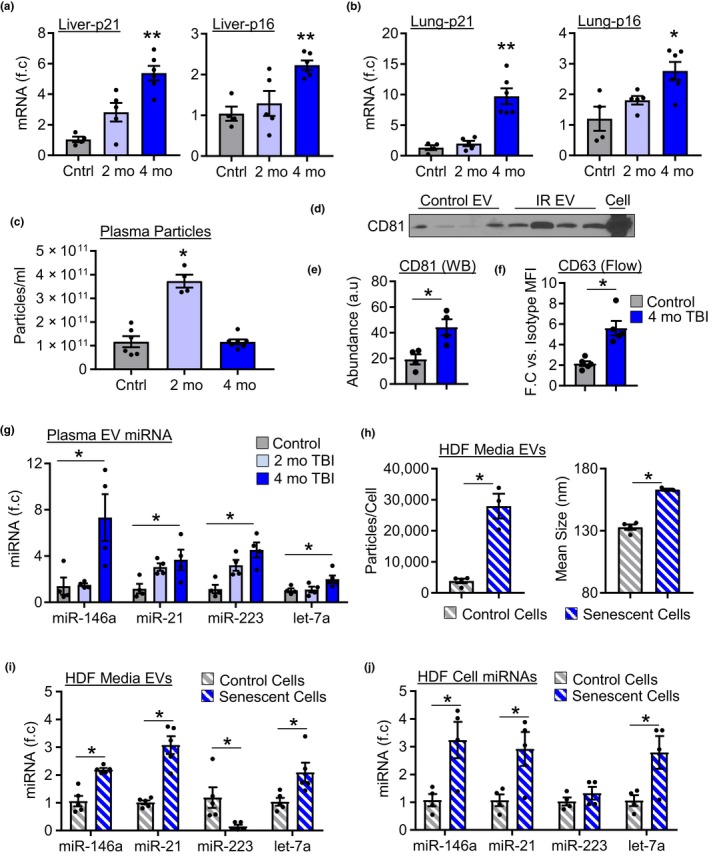
Induction of cellular senescence alters EV secretion and cargo. Expression of p16 and p21 mRNA in the (a) liver and (b) lung of control, 2 and 4 month post‐TBI mice, *n* = 4–6/group, *p < .05 versus control, **p < .05 versus all groups. (c) Quantification of plasma particle concentration by NTA, *n* = 4–7/group, *p < .05 versus control. (d) Representative Western blot image and (e) quantification, *n* = 4/group, *p < .05. (f) CD63 abundance by flow cytometry, *n* = 5/group, *p < .05. (g) Expression of miRNAs in plasma EVs in control, 2‐ and 4‐month post‐TBI mice, *n* = 4/group, *p < .05 4 month TBI versus control. (h) Quantification of EV concentration and size secreted by control and senescent cells by NTA, *n* = 3–5/group. (i) Expression of miRNAs in EVs isolated from control and senescent cells. *n* = 5/group. (j) Expression of miRNAs in control and senescent cells, *n* = 4/group, *p < .05. All values are mean ± *SEM*

In light of these findings, we next examined whether other cellular sources contribute to changes in plasma EVs with aging. Senescent cells accumulate in multiple organs where they contribute to the development of age‐associated diseases. Therefore, using sub‐lethal total body irradiation (TBI) as an in vivo model of global senescence (Le et al., 2010), we investigated whether senescent cells have an effect on plasma EVs. Examination of senescent cell markers p16 and p21 in the liver (Figure 5a) and lung (Figure 5b) revealed a time‐dependent increase in expression, with significantly greater levels by 4 months post‐TBI compared to control mice. Analysis of plasma particles by NTA revealed that TBI was associated time‐dependent changes, as indicated by increased concentration at 2 months and return to baseline levels by 4 months (Figure 5c). Mean size was also reduced by 2 months but returned to control levels by 4 months (Figure S9a). Assessment of plasma EV levels using flow cytometry and Western blotting at 4 months post‐TBI revealed significantly greater CD63 and CD81 levels, respectively, in TBI versus control mice (Figure 5d–f). Next, we examined whether the induction of senescence altered the expression plasma EV‐like particle miRNAs identified in our analysis of young and old mice. Indeed, expression of miR‐146a, miR‐21, miR‐223, and let‐7a exhibited time‐dependent increases with significantly higher expression in 4‐month TBI mice compared to control mice (Figure 5g). In order to further establish that cellular senescence leads to changes in EV miRNA expression, we examined EV production by senescent cells in vitro. Human dermal fibroblasts (HDFs) were irradiated at 20Gy, cultured for 7 days, and senescence confirmed by increased β‐galactosidase staining (Figure S9c) as well as increased expression of p21, IL6, and Ccl2 compared to control cells (Figure S9d). After 7 days, senescent cells were transferred to serum free media to generate conditioned media and EV secretion and cargo was assessed. Similar to what was observed in vivo, senescent HDFs exhibited a significant increase in EV secretion compared to control cells (Figure 5h). However, induction of senescence in vitro led to a significant increase in mean EV size compared to control cells (Figure 5h). Examination of EV miRNA expression revealed that senescent HDFs secrete significantly greater amounts of EV associated miR‐146a, miR‐21, and let‐7a compared to control cell EVs (Figure 5i). Surprisingly, miR‐223 was dramatically reduced in senescent cell EVs compared with control cell EVs. Examination of cellular miRNA expression revealed that induction of senescence in HDFs leads to significantly increased cellular expression of mir‐146a, miR‐21, and let‐7a compared to control cells (Figure 5j). Expression of miR‐223 was not different between senescent and control cells, suggesting that miR‐223 sorting mechanisms may be altered in senescent cells. Collectively, these results demonstrate that induction of cellular senescence alters EV secretion and cargo in vivo and in vitro.

### 2.6 D + Q therapy reduces expression of senescence cell‐associated miRNAs in old plasma EVs

**Figure 6:**
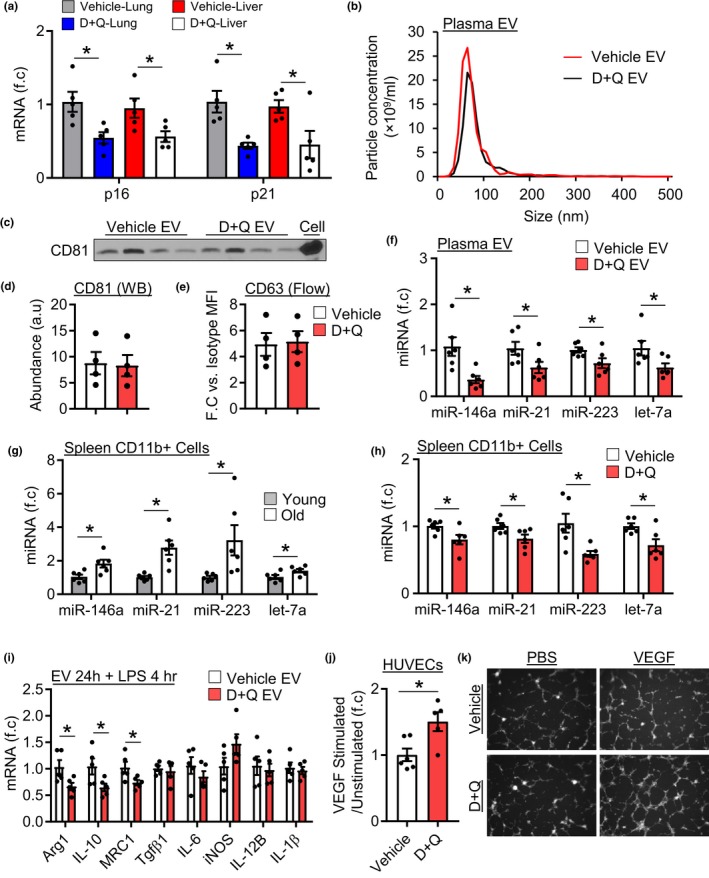
Rejuvenation of old EV‐like particle cargo and function by D + Q treatment. (a) Expression of p16 and p21 in the lung and liver of vehicle and D + Q‐treated mice, *n* = 5/group. (b) Representative size distribution graph from NTA of plasma particles. (c) CD81 Western blot image and (d) quantification, *n* = 4/group, *p < .05. (e) CD63 abundance by flow cytometry, *n* = 5/group, *p < .05. (f) Expression of miRNAs in EVs isolated from vehicle and D + Q plasma, *n* = 6/group, *p < .05. (g) Expression of miRNAs in spleen CD11b^+^ cells isolated from young or old mice, *n* = 6/group. (h) MiRNA expression in CD11b^+^ cells isolated from vehicle or D + Q‐treated old mice, *n* = 6/group. (i) Macrophages treated with vehicle or D + Q plasma EVs then stimulated with LPS, *n* = 5/group. (j) Quantification of tube formation of HUVECs treated with vehicle or D + Q plasma EVs followed by stimulation with VEGF, *n* = 6 for vehicle EV and *n* = 5 for D + Q EV, *p < .05. (k) Representative tube formation images. All values are mean ± *SEM*

In order to establish that senescent cells contribute to plasma EVs with aging, aged mice were treated with an established senolytic combination therapy (Zhu et al., 2015), dasatinib and quercetin (D + Q) or vehicle, biweekly for 2 months. Successful treatment was confirmed by reduced liver and lung *p16* and *p21* mRNA expression in D + Q‐treated mice compared to vehicle‐treated mice (Figure 6a). Examination of plasma particles revealed that D + Q‐treated mice had significantly greater mean particle size compared to vehicle‐treated mice (Figure 6b and Figure S9e). D + Q treatment did not affect overall particle concentration (Figure S9f). Examination of EV levels revealed that D + Q treatment did not affect EV levels, as CD81 and CD63 expression were similar between vehicle and D + Q mice, when examined by Western blotting and flow cytometry, respectively (Figure 6c,d). Comparison of EV‐like particle miRNA expression revealed that D + Q treatment significantly reduced the expression of miR‐146a, miR‐21, let‐7a, and miR‐223 compared to vehicle‐treated mice (Figure 6e). This does not appear to be a generalized effect on EV miRNAs as miR‐145, another miRNA which changed with age, was not affected by D + Q treatment (Figure S9g).

Changes in plasma EV‐like particle miRNAs may lead to changes in cellular miRNA expression. Since myeloid cells are major targets of circulating EVs (Akbar et al., 2017), we compared young and old spleen CD11b cells (myeloid cell marker). CD11b^+^ cells isolated from old mice exhibited significantly greater expression of miR‐146a, miR‐21, miR‐223, and let‐7a compared to young CD11b^+^ cells (Figure 6g). Next, we determined if a reduction in plasma EV‐like particle miRNA levels in D + Q‐treated mice were associated with changes in CD11b^+^ cell miRNA expression. Consistent with changes at the plasma level, D + Q‐treated mice exhibited a significant reduction in miR‐146a, miR‐21, miR‐223, and let‐7a compared to vehicle‐treated mice (Figure 6h), although levels were still elevated above young levels (Figure S9h).

Lastly, we investigated whether reduced expression of senescent cell‐associated miRNAs was associated with functional differences in plasma EV fractions. Macrophages and endothelial cells were treated with plasma EV enriched fractions and tested as before. Treatment of macrophages with D + Q EV fractions led to significantly lower expression of Arg1, IL10, and MRC1 following LPS stimulation compared to cells treated with vehicle EV fractions (Figure 6i). D + Q also restored the effect of old EV fractions on endothelial responses to VEGF, as cells pretreated with D + Q plasma EV fractions but not vehicle exhibited a ~ 1.5‐fold increase in VEGF‐dependent tube formation compared to unstimulated cells (Figure 6j,k). Collectively, these results demonstrate that senescent cells contribute to the circulating plasma EV pool in old mice where they mediate cargo and functional changes. Conversely, removal of senescent cells shifts plasma EV miRNA expression and function toward a younger phenotype.

## 3 DISCUSSION

Circulating plasma vesicles interact with a number of cell types including cells in the bone marrow, liver, and spleen. Although the exact physiological role of plasma EVs is poorly understood, one possible mode of action is that the continuous interaction with recipient cells influences cellular function. Here, we demonstrate that old mice exhibit increased levels of plasma EVs. We also show that miRNAs are differentially expressed in young and old EV‐like particles, and that old EV‐like particles or the miRNAs they carry can alter macrophage responses to LPS, increase macrophage phagocytosis, and reduce endothelial cell responses to VEGF. We show that old plasma particles have greater expression of miR‐146a, miR‐21, miR‐223, and let‐7a, and treatment of macrophages with old EVs leads to increased cellular expression in recipient cells, whereas young particles do not. Mechanistically, these miRNAs are predicted to target key signal transduction factors in the MAPK (Cheng et al., 2013) and AKT (Meng et al., 2007) pathways which play important roles in regulating cellular responses to stimuli. Although changes in these miRNAs occur in immune cell aging, we find that circulating cells do not significantly contribute to changes in these miRNAs, as reconstitution of aged mice with young BM cells did not reduce the expression of age‐associated miRNAs. Alternatively, we identify a role of cellular senescence in regulating circulating EV miRNA levels.

The accumulation of senescent cells plays a major role in the development and progression of age‐related pathologies (Robbins, 2017; Takasugi, 2018). This is due in part to the senescent‐associated secretory profile (SASP) acquired by senescent cells. More recent descriptions of the SASP have been expanded to include the secretion of EVs (Robbins, 2017; Takasugi, 2018). Extracellular vesicles derived from senescent cells exhibit multiple changes in cargo compared to healthy cells including changes in protein (Takasugi et al., 2017), and miRNA abundance (Terlecki‐Zaniewicz et al., 2018). To date, studies have focused on changes in EV cargo and functions in vitro. Using in vitro and in vivo senescence models, we find that the induction of senescence leads to increased expression of miR‐146a, miR‐21, and let‐7a in secreted EVs. We further demonstrate a role of senescent cells in regulating plasma particle miRNA expression using senolytic treatment of aged mice. Through transcriptome analysis of senescent cells Kirkland and colleagues developed a dasatinib and quercetin (D + Q) combination therapy which targets the pro‐survival pathways activated in these cells (Zhu et al., 2015). Here, we show that senolytic therapy using D + Q reduces the expression of age‐associated miRNAs and shifts plasma particle function to a younger phenotype. Moreover, we show that removal of senescent cells affects miRNA expression in splenic CD11b^+^ cells, and these changes correlate with changes in the circulation. Interestingly, D + Q treatment did not reduce plasma EV levels but did reduce miRNA expression. It is possible that senolytics act to suppress the intracellular pathways activated in senescent cells to alter the secreted factors. Further examination into the effect of senolytics on EV secretion and cargo is needed. Furthermore, although we identified and followed changes in miRNA expression throughout the study it is important to note that EVs carry a diverse cargo (e.g., proteins and lipids) which may also play important functional roles. MicroRNAs are likely one of many functional molecules which change with age. Further investigation into the contribution of other components in the EVs is also needed.

The importance of our findings is twofold. First, our data suggest that changes in EV‐like particle miRNA levels can be monitored to gauge the accumulation of senescent cells or efficacy of senolytic therapy. Examination of plasma markers is less invasive compared to tissue markers making EV cargo ideal biomarker candidates. Secondly, plasma EVs can transfer cargo to recipient cells and alter cellular function. Therefore, in conjunction with removal of senescent cells, restoring circulating EV cargo toward that of a younger phenotype may also have physiological benefits, as changes in miRNA expression play key roles in regulating changes in cell function with age. For example, increased miR‐146a expression in aged macrophages limits cellular responses to LPS (Jiang et al., 2012). Elevated miR‐21 can also induce senescence and reduce angiogenic potential in endothelial cells (Dellago et al., 2013) as well as impair T‐cell activation (Kim et al., 2018). Changes in miR‐223 and let‐7a expression have also been shown to affect a number of myeloid cell processes including the response to cell activation (Cho, Song, Oh, & Lee, 2015; M'Baya‐Moutoula et al., 2018). It is possible that continuous exposure to circulating EVs carrying senescence cell derived factors promotes changes in cell function. This is why we chose a pretreatment paradigm as cells throughout the aged body would be continuously exposed to circulating EVs prior to activation over the course of days, months, or years depending on the cell type. Conversely, senolytic therapy may decrease these factors (e.g., miRNAs) reduce exposure and slow the aging process. Consistent with this notion, antagomiRs targeting miR‐146a (Jiang et al., 2012) or miR‐21 (Thum et al., 2008) can improve age‐associated phenotypes. It is important to note that the circulating EV pool is diverse and derived from a number of different cell sources; the senescent signature is only one part of the EV pool. Direct and indirect effects of senolytics are likely detected when examined in vivo. This is illustrated by the expression of miR‐223 in our study. Therefore, senescent cells may also influence plasma EV miRNA expression by controlling the activity of other cell types.

Although we used ~2 × 10^9^–1 × 10^10^ particles/ml to assess the functional effects of plasma EV‐like particles, this does not reflect the number of EVs used. Using cryo‐EM and flow cytometry, Brisson and colleagues estimated EVs in human platelet‐free plasma to be 1–10 × 10^7^ EVs/ml (Arraud et al., 2014). However, the authors comment that estimates of the small EV populations, the most abundant EV sub‐type, were likely underestimated using these techniques. This is consistent with data from other groups which suggest that the isolation and quantification methods have a major impact on plasma EV concentration and purity (Johnsen, Gudbergsson, Andresen, & Simonsen, 2019). By comparing 38 articles published from 2013 to 2018 which isolated plasma EVs using a number of methods, Johnsen and colleagues estimate that the number of circulating plasma EVs in humans is ~1 × 10^10^ particles/ml. However, no current method exists to measure the exact number of EV particles in plasma. Our particle doses of up to 1 × 10^10^ particles/ml does not reflect the number of bona fide EVs, rather a combination of multiple particle types. However, our doses used are not greater than the number of circulating particles at any given time based on our NTA estimates; therefore, our doses are unlikely to be supra‐physiological.

Taken together, the findings in this study demonstrate that senescent cells contribute to the plasma EV pool in old mice. Changes in plasma EV miRNA expression and EV‐like particle functions correlate with the induction and removal of senescent cells in vivo. Collectively, this study suggests a novel mechanism by which senescent cells affect the function of cells throughout the body and contribute to changes in cell function with aging.

## EXPERIMENTAL PROCEDURES

Experimental procedures can be found in supporting information online methods.

## CONFLICT OF INTEREST

None declared.

## AUTHORS' CONTRIBUTION

FJA and RKL conceived and designed the study. FJA, FL, AY, PD, TB, AB, LW, DG, SM, SHL, JEF, and RKL designed and/or performed the experiments. FJA, JEF, RWD, and RKL edited and revised the manuscript, with contribution from all authors.
