# Supplementary figures and images for: Cellular senescence contributes to age‐dependent changes in circulating extracellular vesicle cargo and function
Source: Aging Cell. 2020 Jan 21;19(3):e13103. doi: 10.1111/acel.13103 (PMC7059145; doi:10.1111/acel.13103)

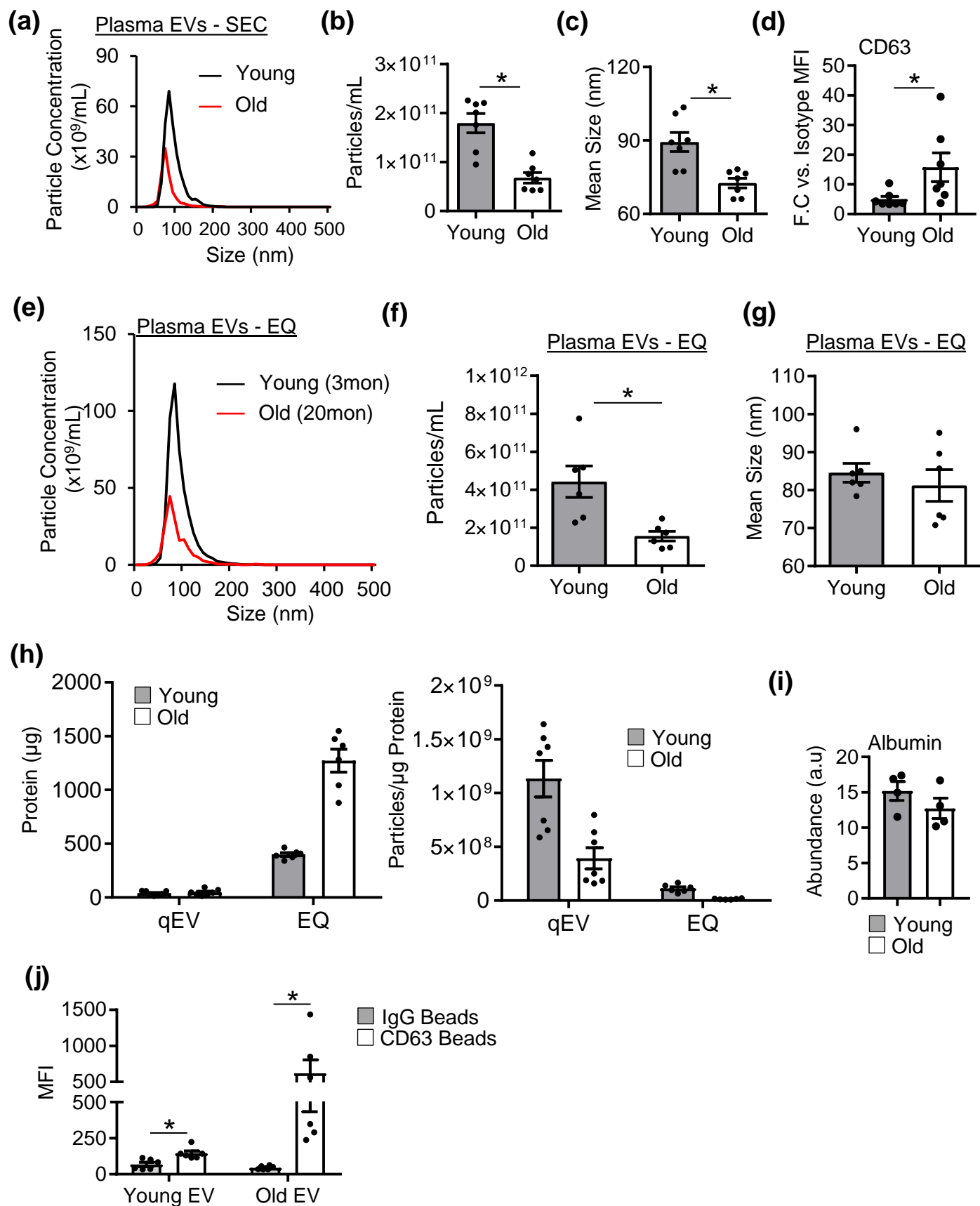

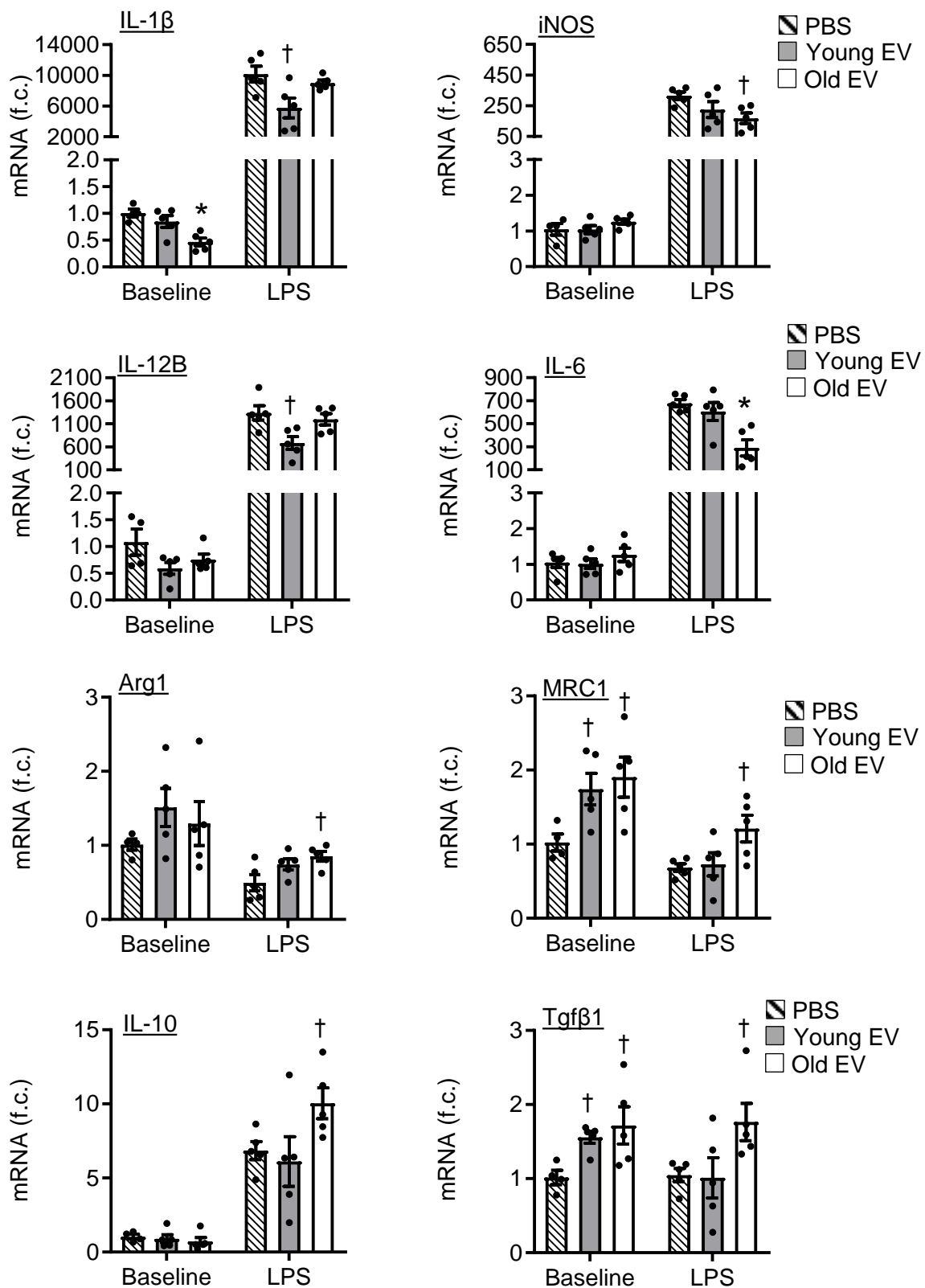

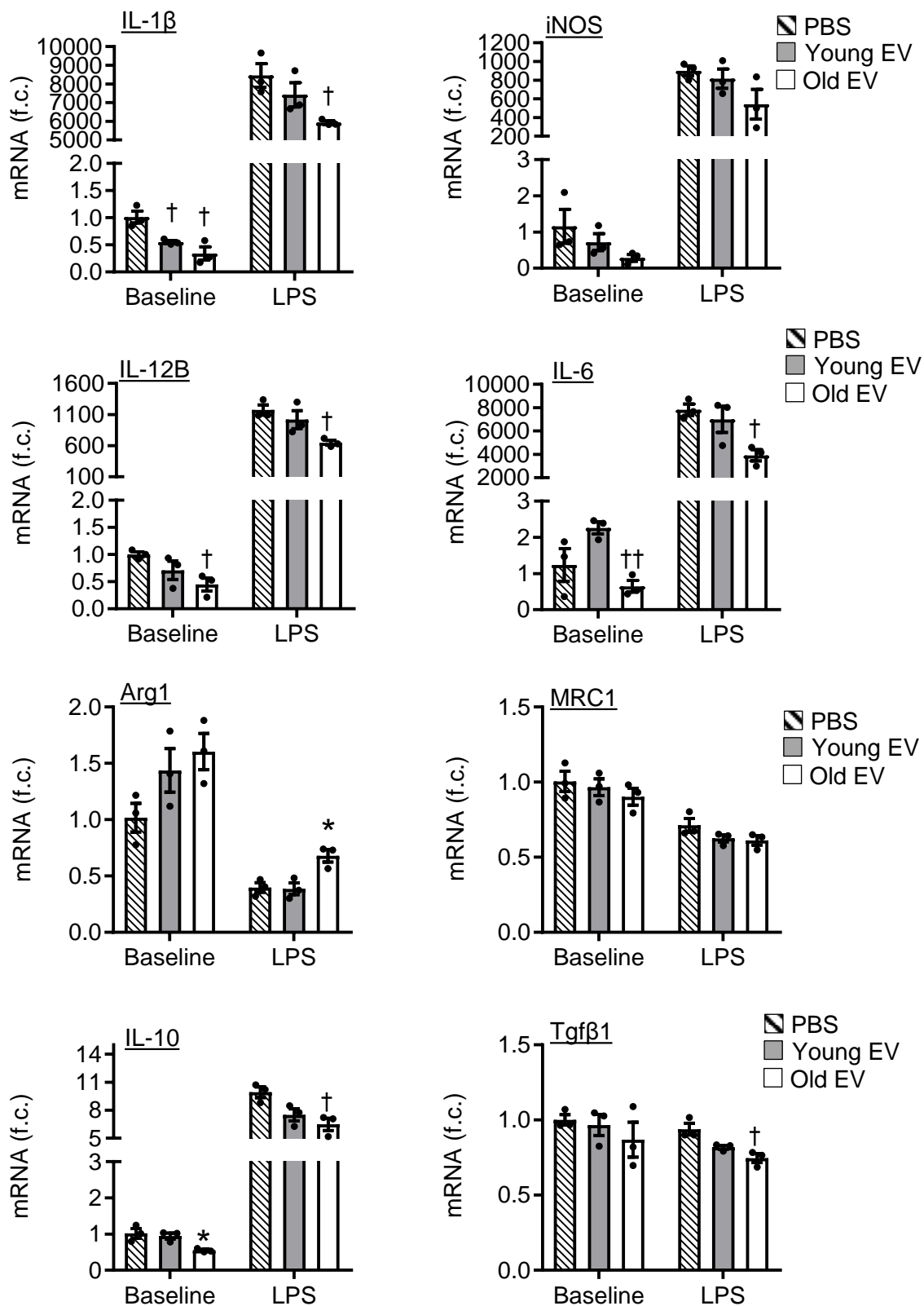

**(a)** Gating Strategy for Peritoneal Macrophage Uptake

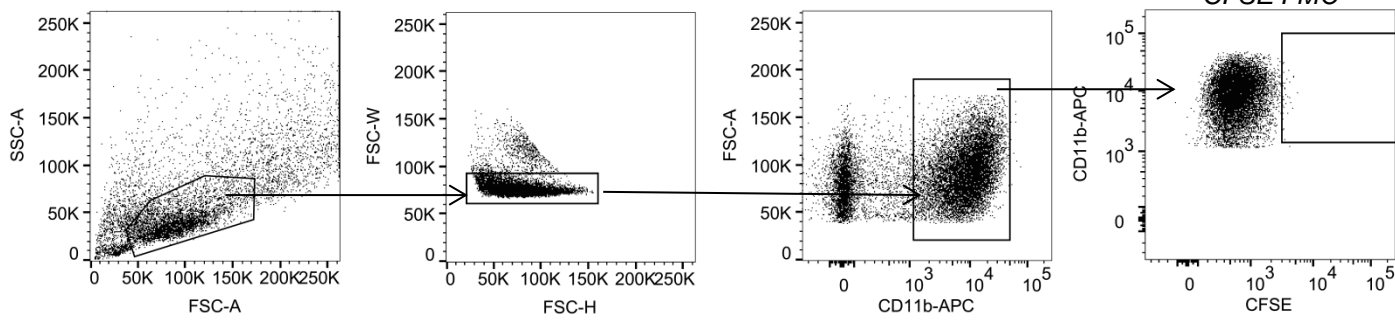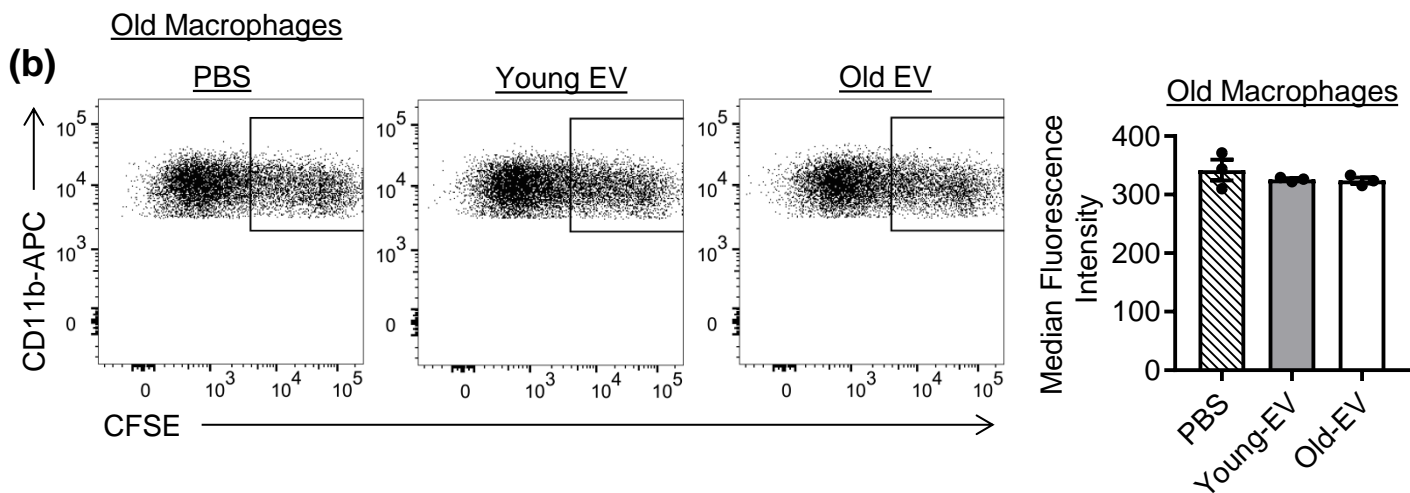

**(c)**

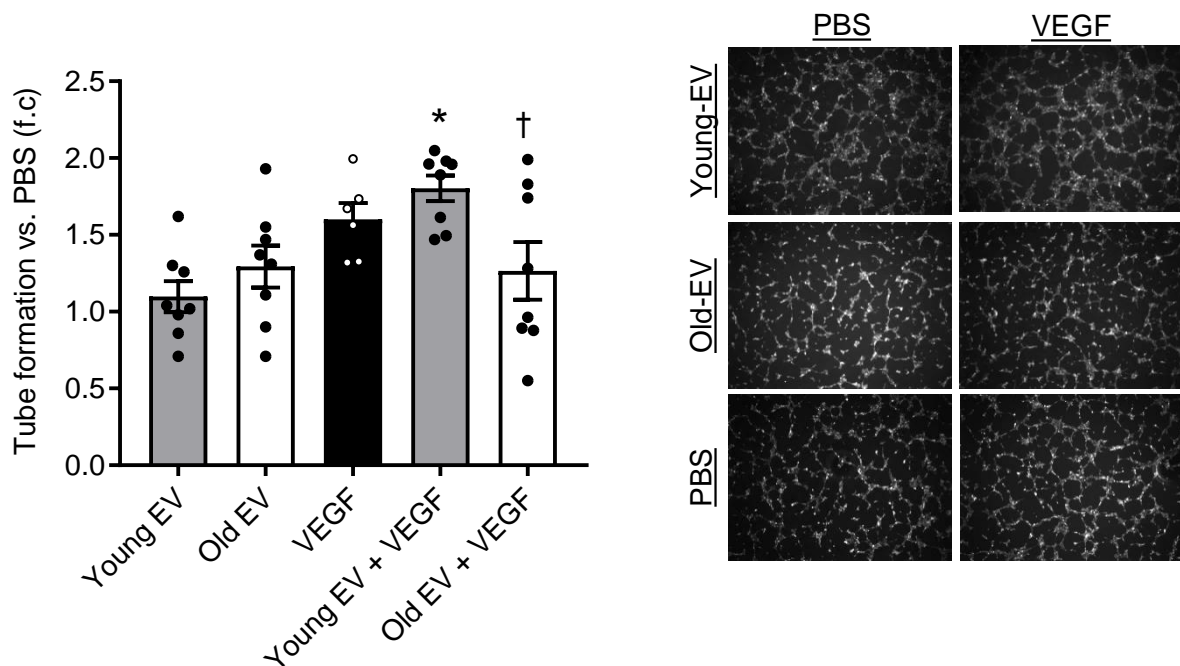

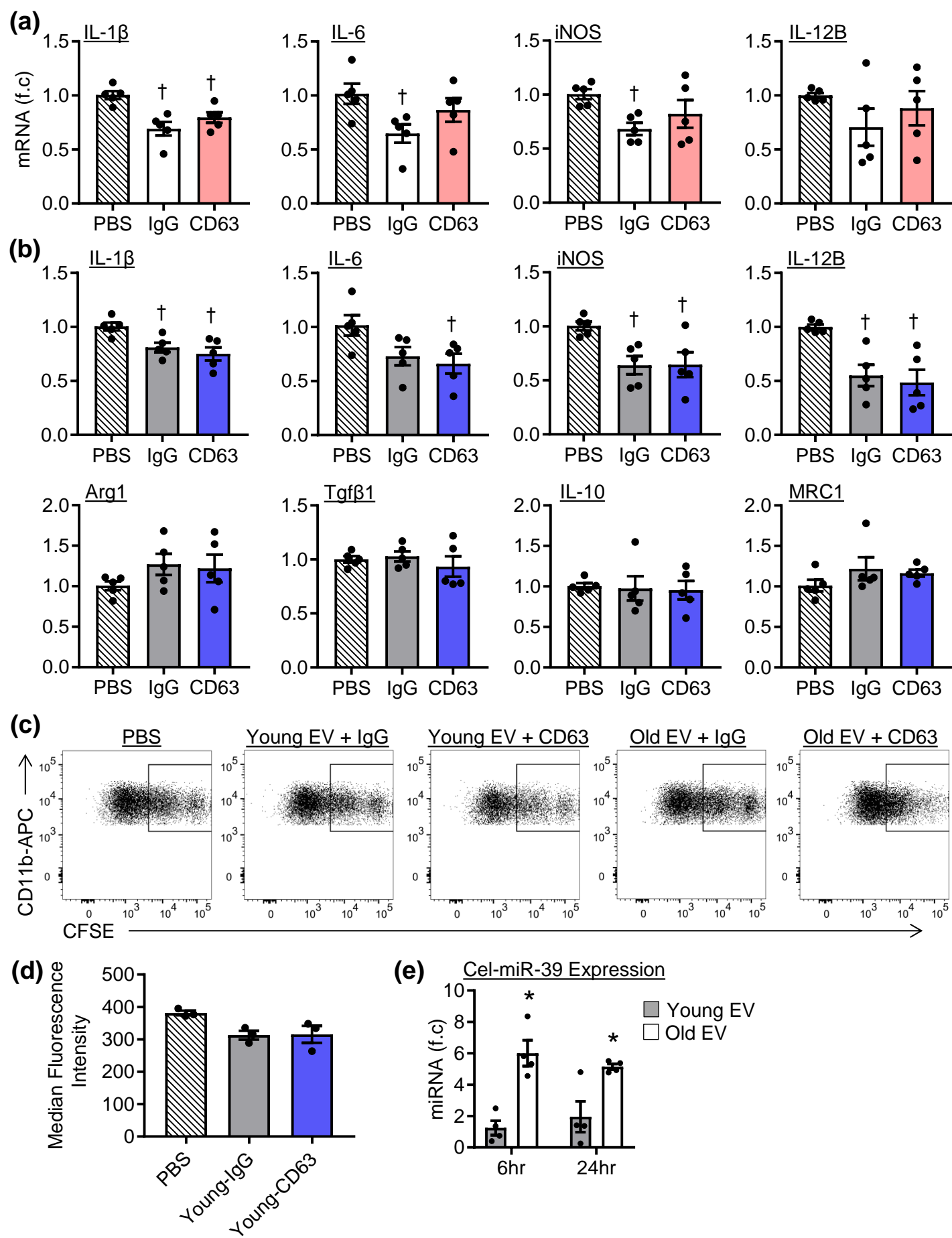

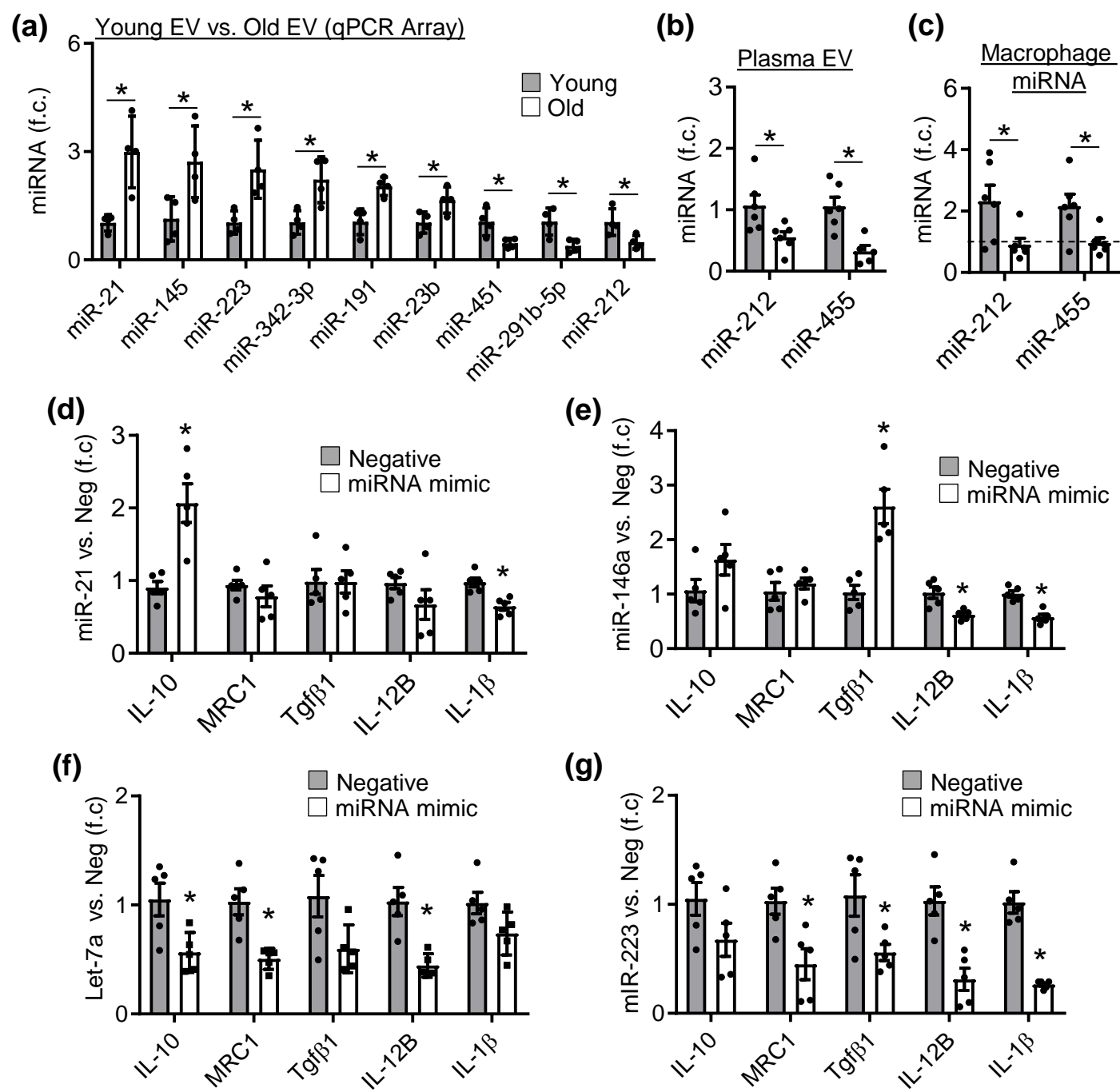

**(a)** Gating Strategy for CD45+/GFP+ Cells

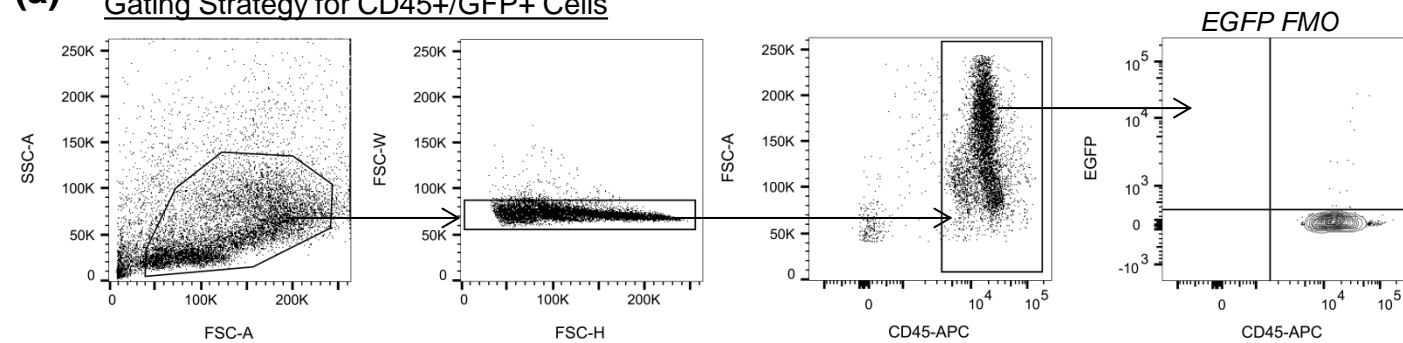

**(b)**

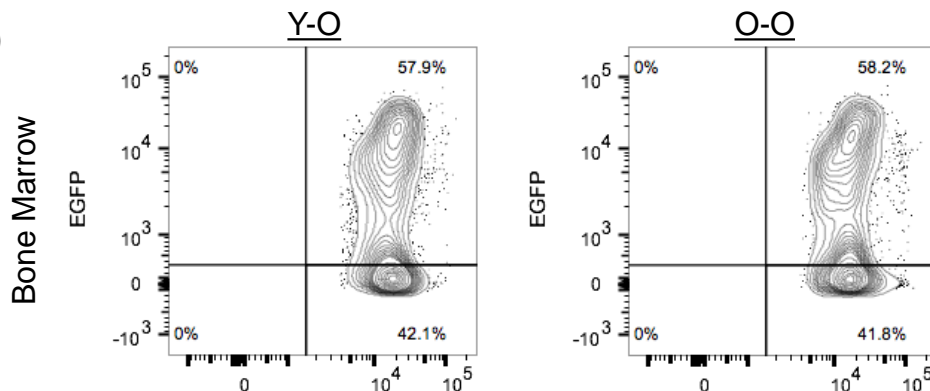

**(c)**

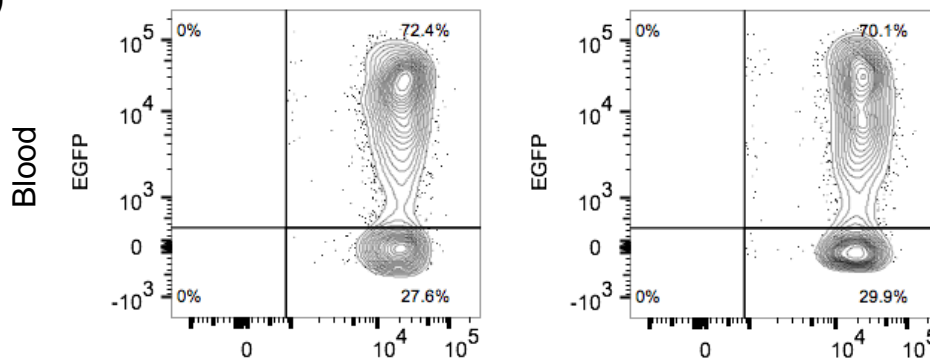

**(d)**

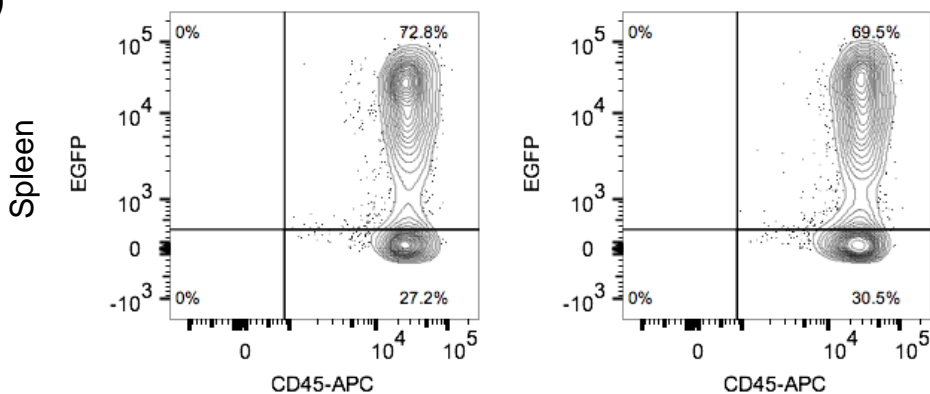

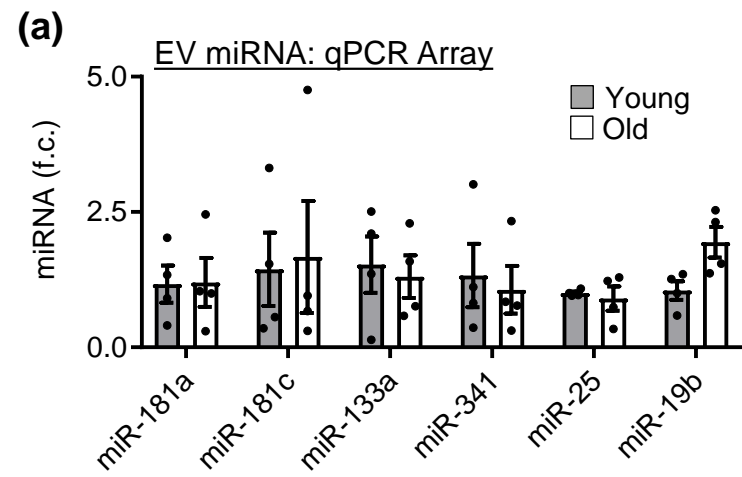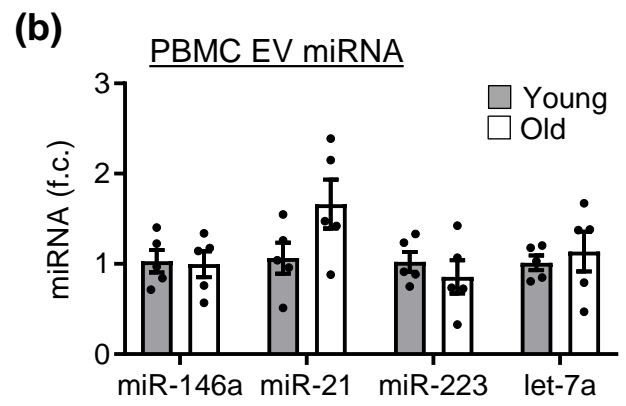

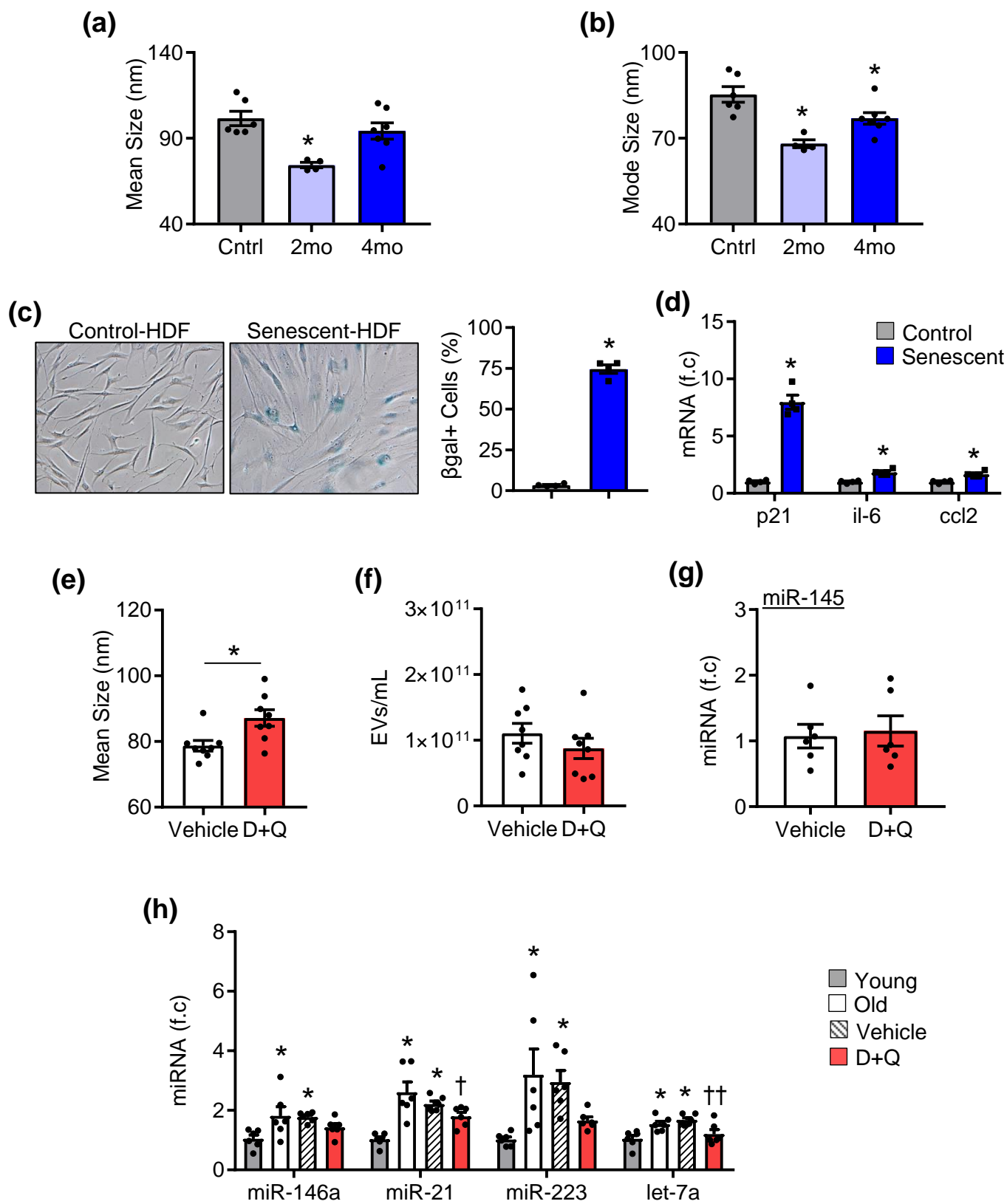

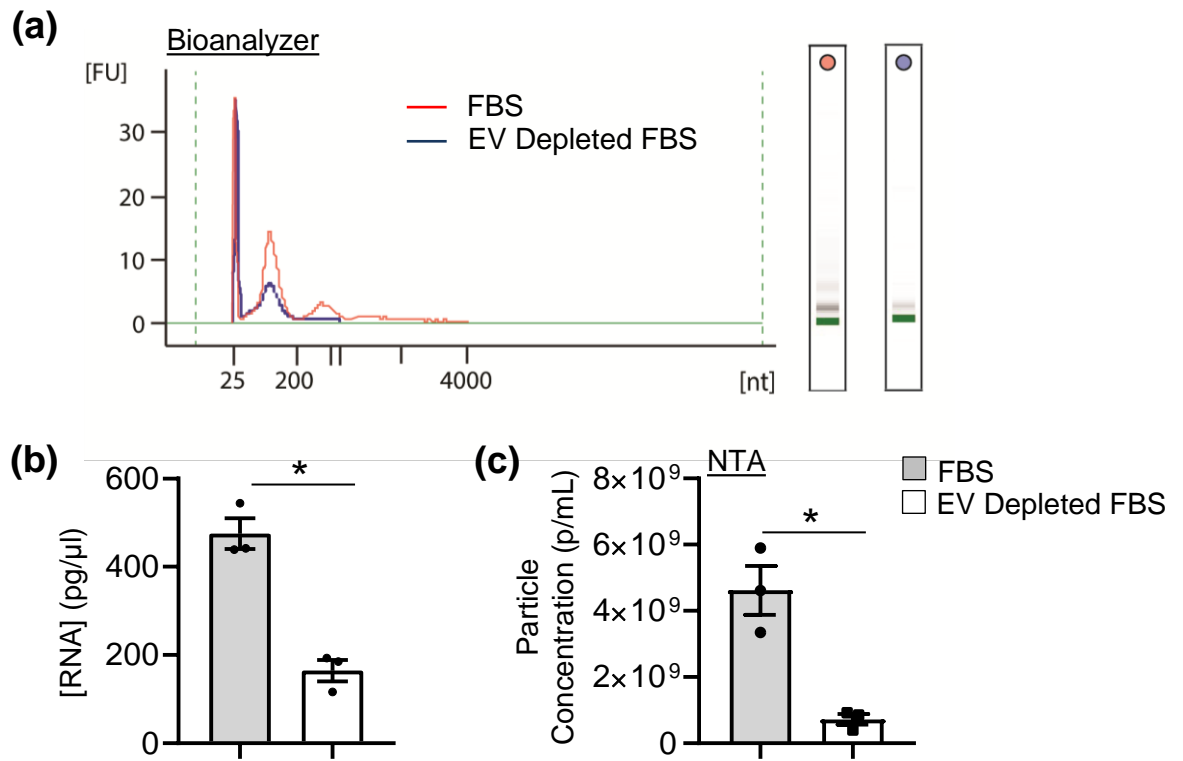

Supplement: Supplementary file 1 [file ACEL-19-e13103-s001.pdf]
