# Supplementary material for: Cellular senescence contributes to age‐dependent changes in circulating extracellular vesicle cargo and function
Source: Aging Cell. 2020 Jan 21;19(3):e13103. doi: 10.1111/acel.13103 (PMC7059145; doi:10.1111/acel.13103)
Supplement: Supplementary file 2 [file ACEL-19-e13103-s002.docx]

**Supporting Information Table 1.** Primer List

| Primer | Product Number | Forward (5’-3’) | Reverse (5’-3’) |
| --- | --- | --- | --- |
| hsa-let-7a-5p | YP00205727 |  |  |
| hsa-mir-223-3p | YP00205986 |  |  |
| hsa-mir-21a-5p | YP00204230 |  |  |
| hsa-mir-146a-5p | YP00204688 |  |  |
| hsa-miR-145-5p | YP00204483 |  |  |
| hsa-miR-16-5p | YP00205702 |  |  |
| hsa-miR-22-3p | YP00204606 |  |  |
| hsa-miR-212-3p | YP00206022 |  |  |
| hsa-miR-455-3p | YP00205432 |  |  |
| cel-miR-39-3p | YP00203952 |  |  |
| U6 snRNA | YP00203907 |  |  |
| hP21 |  | TGTCACTGTCTTGTACCCTTG | GGCGTTTGGAGTGGTAGAA |
| hIL6 |  | CAACCTGAACCTTCCAAAGATG | ACCTCAAACTCCAAAAGACCAG |
| hCCL2 |  | CAGAAGTGGGTTCAGGATTCC | ATTCTTGGGTTGTGGAGTGAG |
| mIL-10 |  | CCAAGCCTTATCGGAAATGA | TTTTCACAGGGGAGAAATCG |
| mMRC1 |  | CTCTGTTCAGCTATTGGACGC | CGGAATTTCTGGGATTCAGCTTC |
| mTGFβ1 |  | CTCCCGTGGCTTCTAGTGC | GCCTTAGTTTGGACAGGATCTG |
| mIL-1β |  | CAGGCAGGCAGTATCACTCA | AGGCCACAGGTATTTTGTCG |
| mIL-12B |  | TGGTTTGCCATCGTTTTGCTG | ACAGGTGAGGTTCACTGTTTCT |
| mP16 |  | CGCAGGTTCTTGGTCACTGT | TGTTCACGAAAGCCAGAGCG |
| mP21 |  | GCCCGAGAACGGTGGAACTT | GACAAGGCCACGTGGTCCTC |
| mArg1 |  | AAGAATGGAAGAGTCAGTGTGG | GGGAGTGTTGATGTCAGTGTG |
| mIL-6 |  | CCGGAGAAGAGACTTCACAG | GGAAATTGGGGTAGGAAGGA |
| miNOS |  | GCAAACATCACATTCAGATCCC | TCAGCCTCATGGTAAACACG |
| mGAPDH |  | CGGCACAGTCAAGGCCGAGAATGG | TCATGGATGACCTTGGCCAGGGGG |
| mHRPT |  | CCCCAAAATGGTTAAGGTTGC | AACAAAGTCTGGCCTGTATCC |
