# Supplementary material for: Cellular senescence contributes to age‐dependent changes in circulating extracellular vesicle cargo and function
Source: Aging Cell. 2020 Jan 21;19(3):e13103. doi: 10.1111/acel.13103 (PMC7059145; doi:10.1111/acel.13103)
Supplement: Supplementary file 3 [file ACEL-19-e13103-s003.docx]

**Supporting Information Table 2**. Analysis of detectable miRNAs in young and old plasma EVs.

| **miRNA** | **p-value** | **Fold Change (O vs Y)** | **95% CI low** | **95% CI high** |
| --- | --- | --- | --- | --- |
| mmu-miR-21 | 0.005 | 2.85 | 1.62 | 5.01 |
| mmu-miR-223 | 0.006 | 2.42 | 1.44 | 4.08 |
| mmu-miR-342-3p | 0.014 | 2.15 | 1.25 | 3.7 |
| mmu-miR-451 | 0.018 | 0.44 | 0.24 | 0.82 |
| mmu-miR-291b-5p | 0.02 | 0.36 | 0.16 | 0.79 |
| mmu-miR-212 | 0.022 | 0.46 | 0.25 | 0.85 |
| mmu-miR-191 | 0.035 | 2.03 | 1.09 | 3.77 |
| mmu-miR-145 | 0.044 | 2.58 | 1.04 | 6.42 |
| mmu-miR-23b | 0.047 | 1.62 | 1.01 | 2.61 |
| mmu-miR-23a | 0.066 | 1.98 | 0.93 | 4.21 |
| mmu-miR-19a | 0.132 | 1.73 | 0.79 | 3.77 |
| mmu-miR-24 | 0.15 | 1.47 | 0.83 | 2.6 |
| mmu-miR-27a | 0.169 | 2.29 | 0.56 | 9.33 |
| mmu-miR-148b | 0.182 | 2.19 | 0.6 | 8.01 |
| mmu-let-7c | 0.189 | 0.42 | 0.1 | 1.76 |
| mmu-miR-92a | 0.228 | 1.44 | 0.72 | 2.87 |
| mmu-miR-126-5p | 0.239 | 0.84 | 0.6 | 1.18 |
| mmu-miR-30d | 0.266 | 1.52 | 0.65 | 3.52 |
| mmu-miR-484 | 0.28 | 0.61 | 0.22 | 1.7 |
| mmu-miR-192 | 0.288 | 1.51 | 0.63 | 3.61 |
| mmu-miR-15b | 0.323 | 1.49 | 0.53 | 4.16 |
| mmu-miR-30b | 0.358 | 1.63 | 0.43 | 6.21 |
| mmu-miR-132 | 0.36 | 1.72 | 0.45 | 6.5 |
| mmu-miR-148a | 0.366 | 1.36 | 0.62 | 3.01 |
| mmu-miR-434-3p | 0.441 | 0.5 | 0.06 | 4.15 |
| mmu-miR-15a | 0.443 | 0.54 | 0.07 | 4.25 |
| mmu-miR-139-5p | 0.521 | 1.34 | 0.46 | 3.9 |
| mmu-miR-101b | 0.542 | 1.83 | 0.12 | 27.97 |
| mmu-miR-30e | 0.587 | 1.16 | 0.62 | 2.15 |
| mmu-miR-215 | 0.602 | 1.4 | 0.26 | 7.59 |
| mmu-miR-30c | 0.613 | 1.19 | 0.53 | 2.65 |
| mmu-miR-20b | 0.667 | 0.94 | 0.67 | 1.32 |
| mmu-miR-574-3p | 0.676 | 1.19 | 0.4 | 3.52 |
| mmu-miR-106a | 0.709 | 1.07 | 0.67 | 1.71 |
| mmu-miR-99b | 0.709 | 0.83 | 0.24 | 2.82 |
| mmu-let-7b | 0.717 | 1.39 | 0.13 | 15.12 |
| mmu-miR-10a | 0.723 | 1.15 | 0.46 | 2.88 |
| mmu-miR-125a-3p | 0.759 | 1.39 | 0.1 | 19.89 |
| mmu-miR-341 | 0.763 | 0.83 | 0.19 | 3.6 |
| mmu-miR-10b | 0.772 | 0.89 | 0.36 | 2.24 |
| mmu-miR-143 | 0.781 | 0.88 | 0.29 | 2.64 |
| mmu-miR-20a | 0.805 | 0.94 | 0.49 | 1.78 |
| mmu-miR-195 | 0.846 | 1.05 | 0.58 | 1.9 |
| mmu-miR-30a | 0.851 | 1.09 | 0.38 | 3.12 |
| mmu-miR-133a | 0.878 | 1.13 | 0.15 | 8.35 |
| mmu-miR-150 | 0.89 | 0.96 | 0.5 | 1.85 |
| mmu-miR-29a | 0.906 | 0.93 | 0.21 | 4.19 |
| mmu-miR-133b | 0.917 | 1.07 | 0.23 | 5.06 |
| mmu-miR-17 | 0.969 | 1 | 0.35 | 1.86 |
| mmu-miR-181c | 0.98 | 0.98 | 0.15 | 6.45 |
| mmu-miR-421 | 0.997 | 1 | 0.15 | 6.91 |

miRNAs above threshold were analyzed using qBase+ to determine the fold change and p-value in expression between young and old plasma EVs. In total 52 miRNAs were above threshold in both groups, miR-16 was detected in all samples and was used to normalize EV miRNA expression.
