# Supplementary material for: Cellular senescence contributes to age‐dependent changes in circulating extracellular vesicle cargo and function
Source: Aging Cell. 2020 Jan 21;19(3):e13103. doi: 10.1111/acel.13103 (PMC7059145; doi:10.1111/acel.13103)
Supplement: Supplementary file 4 [file ACEL-19-e13103-s004.docx]

**Supporting Information Online Methods**

**Experimental** **Procedures**

**Animals**

The Animal Care Committee of the University Health Network approved all experimental procedures which were carried out according to the Guide for the Care and Use of Laboratory Animals (NIH, revised 2011). Female young (3 month) and old (18-21 month) C57Bl/6N mice (Charles Rivers) were used for all experiments. Mice were housed in a 12hr light: 12hr dark cycle with food and water provided *Ad* *Libitum*. All animals were euthanized by isoflurane overdose followed by cervical dislocation.

**Cell Culture**

Human dermal fibroblasts (HDFs) were cultured in DMEM with 10% FBS and 1% pen-strep. Cells were seeded at 5000 cells/cm^2^ and were used for experiments at passage 3-5. For isolation of peritoneal macrophages, young or old mice were injected with 4% thioglycollate and cells isolated by peritoneal lavage 3 days later. Cells were cultured at 1x10^6^ cells/mL in DMEM/F12 supplemented with 1% pen-strep and 10% heat inactivated EV depleted FBS Peripheral blood mononuclear cells were isolated from blood using Ficoll gradient separation. Blood was collected from young and old mice by cardiac puncture into EDTA tubes after which whole blood was layered on a Ficoll Gradient. Following centrifugation interphase cells were collected, washed and counted. Cells were plated at 1x10^6^/mL and cultured in RPMI media with 5% EV depleted FBS and 1% pen-strep. Cells were cultured for 24hrs after which conditioned media was collected and EVs isolated using TEIR (Thermofisher), after 500g, and 3000g spins to remove cellular debris from conditioned media. In order to deplete EVs from fetal bovine serum (FBS), the serum was spun at 120,000g (rcf average) for 18hr at 4°C (SW32 Ti Rotor, k-factor 214), as described by others ([Shelke, Lasser, Gho, & Lotvall, 2014](#_ENREF_4)). Supernatant was removed and filtered through a 0.22µm filter before use. To determine the extent of depletion, EVs were isolated from 1mL of media with no cells containing 10% EV depleted FBS or unmodified FBS using the total exosome isolation reagent. Total particle count was determined using NTA and EV total RNA abundance measured on the Agilent 2100 Bioanalyzer system using the Eukaryote Total RNA Pico Chip (**Figure S10**). Total EV RNA was isolated using the miRNeasy Serum/Plasma Advanced Kit (Qiagen; 217204). For EV treatment 1x10^6^macrophages were treated with 1x10^10^ EVs/mL for 24hrs prior to LPS stimulation. Macrophages were stimulated with LPS (10ng/mL; Sigma, Derived from O111:B4 E. coli) for 4hrs. Human Umbilical Vein Endothelial Cells (HUVECs) were cultured in ECM media supplemented with 5% EV depleted FBS, 1% pen-strep, and Endothelial Cell Growth Supplement (Sciencell). To study activation of endothelial cells, cells were simulated with VEGF (50ng/mL) and tube formation measured after 8hrs as previously described ([Roy et al., 2018](#_ENREF_3)). For EV treatment, 2x10^5^ endothelial cells were treated with 2x10^9^ EVs/mL for 24 hours prior to VEGF stimulation. For both EV treatments the EV:Cell ratio used was 10,000 EVs:Cell. Jurkat T-cells were cultured in complete RPMI (10% FBS and 1% pen-strep). In order to study macrophage phagocytosis Jurkat cells were stained with 2.5µM CFSE (Thermo Fisher) for 15 minutes (37°C) at 2x10^7^ cells/mL in serum free media as described elsewhere ([Celhar et al., 2016](#_ENREF_1)). An equal volume of FBS was added to stop the reaction. Cells were washed and incubated with 30µM etoposide (Sigma) for 18hrs to induce apoptosis. The extent of apoptosis was determined by flow cytometry using the Annexin V Apoptosis Detection Kit (BD Biosciences, 556547) and cells were found to be ~80% Annexin V^+^. Cells were washed three times, re-suspended in DMEM/F12 media (10% EV depleted FBS, 1% pen-strep) and added to cultured peritoneal macrophages at a ratio of 1:1 (apoptotic cells : macrophages) for 1hr. After 1hr cells were placed on ice to inhibit phagocytosis, washed with PBS to remove remaining cells were collected and stained on ice with specific antibodies. The percentage of CFSE-positive macrophages was used as a measure of phagocytosis.

**Extracellular Vesicle Isolation and Characterization**

For isolation of EVs from the plasma, blood was collected by cardiac puncture in to EDTA coated tubes and centrifuged for 3,000g for 15 minutes twice, after which plasma was stored at -80°C. EVs were isolated from plasma using the qEV original size exclusion chromatography columns (Izon) or ExoQuick (System Biosciences) according to the manufacturer’s instructions. Following isolation by size exclusion chromatography the vesicle enriched elution was concentrated using Amicon 10K MWCO centrifugal filters (Millipore). Protein concentration was determined by measuring absorbance at 280nm using the Nanodrop ND1000 (Thermo Fisher). For isolation from conditioned media, cells were incubated in serum free media for 48hrs after which the media was centrifuged at 500g for 15 minutes then 3,000g for 15 minutes at 4°C, each time removing the supernatant. The media was filtered using a 0.22µm syringe filter then concentrated then by ultrafiltration (UF) using the Amicon 10K MWCO filters (Millipore). Following UF, vesicles were isolated using the total exosome isolation reagent (TEIR; Invitrogen). EVs concentration and size distribution was determined by nanoparticle tracking analysis (NTA) using the Nanosight LM10 or NS300 using an automatic syringe pump (Harvard Apparatus) with speed set to 50. Data were collected and analyzed using Nanosight NTA software v3.2 (Malvern Instruments). Data were collected using a green laser (532nm), camera shutter set to 1259 and camera gain to 366. Three videos from each sample were recorded for particle tracking analysis with threshold set to 4. Transmission electron microscopy analysis was performed by the nanoscale Biomedical Imaging Facility at the Hospital for Sick Children using a method previously described by others ([Thery, Amigorena, Raposo, & Clayton, 2006](#_ENREF_5)). Vesicles were fixed in 2% PFA and placed on Formvar-carbon coated EM grids for 2hrs. Grids were washed once with PBS and fixed in 2.5% glutaraldehyde for 10 minutes. Grids were washed with water then transferred to a uranyl-oxalate solution (pH 7) for 5 min. Grids were transferred to methyl cellulose-UA for 10min on ice after which excess solution was dried with a filter paper and grids air dried before imaging. In order to assess EVs by flow cytometry, CD63 coated latex beads were used to capture plasma EVs. Aldehyde/Sulfate 4µm beads (Thermo Fisher) were coated with anti-mouse CD63 (Biolegend: 143901), or Rat IgG2ak Control (Biolegend: 400501) in MES buffer (0.025M, pH 6.0). Coated beads were added to SEC purified EV fractions and incubated overnight at 4°C in capture buffer (0.1% BSA-PBS). The following day unoccupied sites were blocked with glycine (100mM) for 30 minutes at room temperature and washed in capture buffer using a 4,500g 15 minute spin. EVs treated with IgG or CD63 capture beads were divided into two tubes and stained with Rat IgG2ak-PE (Biolegend:400507) control or CD63-PE (Biolegend: 143903) for 30 minutes on ice. Beads were washed and at least 10,000 events collected on a LSR II flow cytometer. To quantify CD63 levels the mean fluorescence intensity (MFI) of CD63 stained beads was dived by MFI of the IgG stained beads. For EV depletion studies, EVs were incubated with CD63 or IgG coated beads overnight at 4°C in PBS. The following day beads were removed by centrifugation, and supernatant used for functional studies as described above.

**MicroRNA and mRNA qPCR**

Total RNA was purified from plasma EVs using two methods. For qPCR based miRNA screening total RNA was isolated using the SeramiR Exosome RNA Isolation Kit (RA800A-1; System Biosciences). RNA was reverse transcribed using the SeramiR Reverse transcription kit (RA800A-1) and miRNA expression assessed using the SeramiR Mouse Profiler Kit (RA810A-1) on a 384 well plate on a LightCycler 480 (Roche). Cycling parameters were according to the manufacturer’s instructions: 50°C 2min, 95°C 10min, (95°C 15s, 60°C 1min)x 40 cycles with data read at the end of the 60°C cycle. For array analysis plasma from 2 mice were pooled for each isolation yielding an n=4/group from a total of 8 mice/group. Stably expressed miRNAs for normalization were assessed using GeNorm and differential expression of miRNAs determined using qBase+ (Biogazelle) with a fold change cut off of ≥1.5. Using this method we identified 4 stably expressed reference genes (M<0.1, miR-126, miR-16, miR-150, and miR-195. MicroRNAs at ≤Ct 35 in at least three samples were considered detectable (above threshold). For all other experiments total EV RNA was isolated using the miRNeasy Serum/Plasma Advanced Kit (Qiagen). RNA was reversed transcribed using the miCURY LNA RT Kit (339340; Qiagen) using an equal volume of RNA. qPCR was performed using miRNA LNA primers as outline in Table S1 SYBR green (ThermoFisher; 4309155) with the following cycling parameters: 95°C 2min, (95°C 15s, 60°C 1min)x 40 cycles with data read at the end of the 60°C cycle. For RNA isolation controls cel-miR-39 was spiked into the solution with isolated EVs prior to RNA isolation. For reverse transcription controls UniSp6 was spiked in during reverse transcription. Lastly, miR-16 was detected in all samples and used as a positive control. Expression levels in EVs were normalized to miR-16. For profiling of miRNAs from cells total RNA was isolated using the miRNeasy kit and miRNAs and expression was normalized to U6. Total RNA was isolated from tissues using QIAzol Lysis Reagent (Qiagen). cDNA was prepared using NxGen M-MulV Reverse Transcriptase (Lucigen) and random primers (ThermoFisher). Gene expression was assessed by quantitative real time-PCR, performed using SensiFAST (Bioline) SybrGreen 2x master mix. All data were analyzed using the ΔΔCT method normalized to the house keeping gene Hrpt or Gapdh. Primers used are provided in **Table S1**.

**Bioinformatics Analysis**

Predicted target mRNAs of differentially expressed miRNAs was determined using miRDIP as described previously ([Tobin et al., 2019](#_ENREF_6)). Only targets present within 5 databases were considered and for increased stringency the top two-thirds of targets were chosen as described previously. Predicted mRNA-miRNA networks were loaded into cytoscape and GO ontology terms determined and clustered based on molecular function using BinGO. Similar GO terms are clustered to identify the molecular functions enriched. Node size is proportional to the number of genes in the GO term and colour proportional to the p-value as indicated in the heat map legend. KEGG pathways targeted by differentially expressed miRNAs were determined using miRPath (v3) using the gene union function, microT-CDS (v5.0) prediction, microT threshold of 0.8, and p-value cut off of p<0.00001 with Benjamini & Hochberg false discovery rate.

**Senescence Assays**

HDFs were irradiated at 20Gy and cultured for 7 days in complete media (DMEM with 1% penicillin-streptomycin and 10% fetal bovine serum). After 7 days senescence was confirmed by assessing β-Galactosidase activity (Cell Signalling) and by changes in cell mRNA expression. For conditioned media experiments, cells were cultured in complete media for 7 days after irradiation and then switched to serum free media for 48 hours to produce conditioned media. To induce senescence *in-vivo*, mice (2 month old) were non-lethally irradiated (6.0Gy) and after 2 or 4 months tissues and plasma were collected. In order to selectively remove senescent cells in aged mice 19 month old mice were treated with Dasatinib (Sigma) + Quercetin (Sigma) (D+Q) combination therapy, which can effectively remove senescent cells *in-vivo* ([Zhu et al., 2015](#_ENREF_7)). Mice were treated orally with D + Q (5mg/kg and 50mg/kg respectively) or vehicle (10% PEG) bi-weekly for 2 months.

**Bone Marrow Reconstitution**

Bone marrow was isolated from the tibiae and femur of young (3 months) or old (18 month) C57BL/6-Tg (CAG-EGFP) 1Osb/J EGFP mice. Sca-1^+^ cells were enriched using the Sca-1 magnetic purification kit (Stem Cell Technologies) according to the manufacturer’s instructions. Old (18 month) or young (2 month) mice were lethally irradiated at 10Gy using a Gammacell 40 Extractor Cesium-137 Irradiator (Best Theratronics) and were administered 2x10^6^ young or old GFP^+^/Sca-1^+^­ cells intravenously (tail vein). Three months after reconstitution, repopulation of the blood, spleen and bone marrow were assessed by flow cytometry, and plasma EVs were examined as described above.

**Flow Cytometry**

For assessment of macrophage phagocytosis after washing cells were lifted with short trypsin incubation (0.05%, 5min) and pipetting. Cells were washed and stained with CD11b (M1/70, Biolegend) for 20 minutes on ice. Cells were washed and the number of CD11b+/CFSE+ macrophages determined using an LSR II flow cytometer equipped with a violet laser (25mW), blue laser (100 mW), and red laser (20mW). Populations were determined based on FMOs and 10,000 CD11b+ events were collected per sample. Sample gating is shown in **Figure S4A.** For flow cytometry of bone marrow single cell suspensions were obtained by flushing the bone marrow with a 23 gauge needle, breaking up the plug by trituration with a pipette tip and 2 passes through an 18 gauge needle. Red blood cells were lysed for 5 minutes at room temp using ACK buffer, washed and filtered through a 40µm filter. Cells were blocked using anti-CD16/32 (1:100, Biolegend) and stained with CD45 (1:200, Biolegend; Clone: 30-F11) for 20 minutes on ice. Cells were washed and run on a LSRII equipped with a violet laser (25mW), blue laser (50mW), yellow laser (100mW), and red laser (40mW). At least 10,000 events were collected per sample. All gating was determined by FMOs and donor cells in the blood, spleen and bone marrow were identified as CD45^+^/GFP^+^ cells. Gating used is shown in **Figure S7A.** All data were analysed in FlowJo (TreeStar).

**Western Blotting**

EVs were isolated by SEC as described above and concentrated using the 10K MWCO Amicon Filters (EMD Millipore), ensuring that final volumes were equal. For all western blots equal volume was used to standardize the quantity loaded. 5x RIPA was added to the concentrated EV fractions and lysed on ice for 10 min. Samples were spun down at 10,000g for 5 min and stored at -20°C until use. 10ug of cardiac fibroblast cell lysate was used as a cell control for all westerns. Reducing conditions were used for all antibodies with exception of CD63. 5x loading buffer was added to samples (with and without β-mercaptoethanol) and heated at 95°C for 5min (all targets except CD63) or 70°C for 10 min (CD63). Samples were separated by SDS-PAGE (10% gel for all targets expect APOB, 5% gel for APOB) and transferred to a nitrocellulose membrane. All targets were transferred for 70 min at 100V, except APOB which was transferred at 20V overnight in a cold room. Membranes were blocked with 5% non-fat milk in TBS-T (0.1% Tween-20) for 1hr and incubated with primary antibodies overnight in non-fat milk or 5% BSA according to manufacturer’s instructions. Primary antibodies and concentrations used were: anti-CD63 (NVG2, Biolegend; 1:500), anti-TSG101 (NBP1-80659, Novus Biologicals; 1:500), anti-CD81 (D5O2Q, Cell Signalling, 1:1000), anti-Albumin (A0353, Abclonal, 1:2000), anti-APOA1 (A1129, Abclonal, 1:1000), anti-Calnexin (NB100-1965, Novus Biologicals, 1:1000), and anti-APOB (NB200-527, Novus Biologicals; 1:1000). Membranes were washed and incubated with either anti-rabbit-HRP (1:2500, Cell Signalling), or anti-rat-HRP (1:2500, Cell Signalling) in 5% non-fat milk TBS-T for 1hr at room temp. Membranes were developed using Super Signal West Pico PLUS ECL reagent (Thermo Fisher) and band intensity quantified using Image J.

**CD11b^+^ Isolation**

CD11b^+^ cells were isolated from spleens by magnetic activated cell sorting (MACS). Single cell suspensions were collected by passing spleens through a 40µm cell strainer. RBCs were lysed, cells resuspended in medium buffer and cells isolated according to the manufacturer’s instructions (Stem Cell Technologies). After the last wash cells were pelleted, re-suspended in Qiazol, and total RNA isolated using the miRNeasy kit (Qiagen).

**MicroRNA Transfection**

Macrophages were transfected with 20nM miScript microRNA mimics (Qiagen) or 20nM Allstars Negative Control (Qiagen). 48 hours following transfection, cells were stimulated with LPS for 4 hours after which cells were collected in Qiazol. For transfection of HUVECs, cells were plated at 50% confluency on fibronectin (10 µg/ml) coated plates. After 24hrs cells were transfected with 20nM miRNA mimic or 20nM Allstars Negative Control using RNAiMax (Invitrogen). Cells were used for tube formation assays 48 hours later. All mimics were synthesized by Qiagen. The following were used: Syn-miR-146a-5p, Syn-miR-21a-5p, Syn-miR-223-3p, and Syn-let-7a-5p. To transfect plasma EVs the Exo-fect siRNA/miRNA transfection kit was used (SBI; EXFT200A-1). EVs were isolated by SEC and concentrated to an equal volume of 100µl in PBS using a 10K Amicon centrifuge filter. EVs were transfected with 1.5µl of cel-miR-39 (10µM Stock, Qiagen) and incubated at 37°C for one hour according to the manufacturer’s instructions. Free miRNA was removed using the provided clean up columns and equal volume (30µl) of transfected EVs added to a 24 well of young peritoneal macrophages. Cells were collected 6hr and 24hr later, total RNA isolated using the miRNeasy kit (Qiagen), and cellular cel-miR-39 expression analyzed. Expression of cel-miR-39 was normalized to U6.

**Statistics**

All values presented are mean ± standard error of the mean (SEM). For comparison of two groups a two tailed unpaired Student’s *t*-test was used. For more than 2 groups, a one way ANOVA was used followed by a Bonferroni post hoc. All data was analyzed in Prism 7 (GraphPad, InStat) unless otherwise stated. MicroRNA expression from the qPCR array was analyzed using the qbase+ software (Biogazelle) using an unpaired two-tail Student’s *t*-test. Principle Component Analysis was performed using ClustVis using unit variance scaling, the default singular value decomposition method, and 0.9 confidence level for ellipses ([Metsalu & Vilo, 2015](#_ENREF_2)). Values of p<0.05 were considered statistically significant.

**References**

Celhar, T., Pereira-Lopes, S., Thornhill, S. I., Lee, H. Y., Dhillon, M. K., Poidinger, M., . . . Fairhurst, A. M. (2016). TLR7 and TLR9 ligands regulate antigen presentation by macrophages. *Int Immunol, 28*(5), 223-232. doi:10.1093/intimm/dxv066

Metsalu, T., & Vilo, J. (2015). ClustVis: a web tool for visualizing clustering of multivariate data using Principal Component Analysis and heatmap. *Nucleic Acids Res, 43*(W1), W566-570. doi:10.1093/nar/gkv468

Roy, A. R., Ahmed, A., DiStefano, P. V., Chi, L., Khyzha, N., Galjart, N., . . . Delgado-Olguin, P. (2018). The transcriptional regulator CCCTC-binding factor limits oxidative stress in endothelial cells. *J Biol Chem, 293*(22), 8449-8461. doi:10.1074/jbc.M117.814699

Shelke, G. V., Lasser, C., Gho, Y. S., & Lotvall, J. (2014). Importance of exosome depletion protocols to eliminate functional and RNA-containing extracellular vesicles from fetal bovine serum. *J Extracell Vesicles, 3*. doi:10.3402/jev.v3.24783

Thery, C., Amigorena, S., Raposo, G., & Clayton, A. (2006). Isolation and characterization of exosomes from cell culture supernatants and biological fluids. *Curr Protoc Cell Biol, Chapter 3*, Unit 3 22. doi:10.1002/0471143030.cb0322s30

Tobin, S. W., Alibhai, F. J., Lee, M. M., Yeganeh, A., Wu, J., Li, S. H., . . . Li, R. K. (2019). Novel mediators of aneurysm progression in bicuspid aortic valve disease. *J Mol Cell Cardiol, 132*, 71-83. doi:10.1016/j.yjmcc.2019.04.022

Zhu, Y., Tchkonia, T., Pirtskhalava, T., Gower, A. C., Ding, H., Giorgadze, N., . . . Kirkland, J. L. (2015). The Achilles' heel of senescent cells: from transcriptome to senolytic drugs. *Aging Cell, 14*(4), 644-658. doi:10.1111/acel.12344

**Supporting Information Figure Legends**

**Supporting Information Figure 1**. (a) Size distribution graphs of particles isolated by size exclusion chromatography (SEC) measured by nanoparticle tracking (NTA). Quantification of SEC purified plasma particle (b) concentration and (c) mean size by NTA, n=7/group, *p<0.05 young vs. old. (d) Flow cytometry analysis of CD63 abundance in plasma EV fractions, n=7/group, *p<0.05. (e) Size distribution of plasma EVs isolated using Exoquick (EQ). (f) Particle quantification and (g) mean size by nanoparticle tracking analysis, n=6/group. (h) Amount of protein measured by absorbance at 280nm in qEV (size exclusion chromatography) and Exoquick (precipitation; EQ) preparations and particles/µg of protein, n=6-7/group. (i) Quantification of albumin from western blot shown in Figure 1d, n=4/group. (j) Staining for CD63 on IgG and CD63 beads showing capture of CD63 particles in young and old plasma. Values are mean ± SEM

**Supporting Information Figure 2.** Real time PCR analysis of young peritoneal macrophage gene expression at baseline and following 4hr of LPS treatment. Cells were pre-treated with PBS, young EVs or old EVs. n=4-5/group, †p<0.05 vs PBS and *p<0.05 vs. all other groups. Values are mean ± SEM

**Supporting Information Figure 3.** Real time PCR analysis of old peritoneal macrophage gene expression at baseline and following 4hr of LPS treatment. Cells were pre-treated with PBS, young EVs or old EVs. n=3/group, †p<0.05 vs PBS, ††p<0.05 vs. YEV, and *p<0.05 vs. all other groups. Values are mean ± SEM

**Supporting Information Figure 4.** (a) Flow cytometry gating strategy used to identify CFSE+ myeloid cells. (b) Representative flow cytometry image of old peritoneal macrophage phagocytosis of CFSE labelled apoptotic cells and quantification (right).n=3/group. (c) Quantification of HUVEC tube formation relative to PBS treated cells and representative images (right).n=6-8/group, *p<0.05 vs. Young EV and †p<0.05 vs. Young EV + VEGF by one way ANOVA followed by Tukey post hoc. Values are mean ± SEM

**Supporting Information Figure 5.** (a) Young peritoneal macrophage gene expression following LPS stimulation. Cells were pre-treated with old EVs previously incubated with IgG or CD63 beads. (b) Young peritoneal macrophage gene expression following LPS stimulation. Cells were pre-treated with old EVs previously incubated with IgG or CD63 beads. n=5/group, †p<0.05 vs. PBS. (c) Representative flow cytometry image of macrophage phagocytosis assay. Cells were treated with young and old EVs previously incubated with IgG or CD63 beads. (d) Quantification of phagocytosis of cells treated with young EVs. n=3/group. (e) Young and old EVs were transfected with cel-miR-39 and young peritoneal macrophages were treated with transfected EVs. Data are normalized to U6 expression and relative to young EV 6hr. n=4/group, *p<0.05 vs. young at same time point by two way ANOVA followed by Tukey post hoc. Values are mean ± SEM

**Supporting Information Figure 6.** (a) Differentially expression miRNAs identified by qPCR array. List is shown in Figure 3A. n=4/group, *p<0.05. (b) Validation of increased expression of miR-212 and miR-455 in young plasma EVs by qPCR. miR-455 was only detected in 5 of 6 old samples. (c) Macrophage expression of miR-212 and miR-455 following treatment with young (grey) or old (white) EV fractions, expression is relative to PBS treated cells (dashed line). Peritoneal macrophages were transfected with (d) miR-21, (e) miR-146a, (f) Let-7a, or (g) miR-223 microRNA mimics after which cells were stimulated with LPS for 4hrs. Fold change values are normalized to transfection control cells, n=5/group. Values are mean ± SEM

**Supporting Information Figure 7.** (a) Flow cytometry gating strategy used to identify GFP+/CD45+ donor cells and representative flow cytometry images from (b) bone marrow, (c) blood, and (d) spleen.

**Supporting Information Figure 8.** (a) QPCR array expression of miRNAs in young and old EVs that were differentially expressed between OO and YO mice, n=4/group. (b) Expression of EV miRNAs secreted by peripheral blood mononuclear cells (PBMCs) in-vitro. n=5/group. Values are mean ± SEM

**Supporting Information Figure 9**. Nanoparticle tracking analysis of (a) mean particle size and (b) mode size in control, 2 mon and 4 mon total body irradiated mice. (c) Representative beta-galactosidase staining in control and senescent human dermal fibroblasts (HDFs) and quantification, n=4/group. (d) Expression of senescent markers in control and senescent cells, n=4/group. Nanoparticle tracking analysis of (e) mean particle size and (f) concentration from EV fractions collected from the plasma of vehicle and D+Q mice. (g) Expression of miR-145 in plasma-EVs of aged mice treated with vehicle or D+Q, n=6/group. (h) CD11b+ miRNA expression in young, old, old vehicle, and old D+Q mice. n=6/group, *p<0.05 vs. young, †p<0.05 vs. old, and ††p<0.05 vs. vehicle. Values are mean ± SEM

**Supporting Information Figure 10**. (a) Bioanalyzer plot showing RNA species size identified in EVs isolated from FBS and EV depleted FBS. (b) Quantification of EV RNA content in FBS and EV depleted FBS, n=3/group, *p<0.05. (c) Nanoparticle tracking analysis of particle concentration of FBS and EV depleted FBS, n=3/group, *p<0.05. Values are mean ± SEM
